# Sperm DNA methylation epimutation biomarker for paternal offspring autism susceptibility

**DOI:** 10.1186/s13148-020-00995-2

**Published:** 2021-01-07

**Authors:** Nicolás Garrido, Fabio Cruz, Rocio Rivera Egea, Carlos Simon, Ingrid Sadler-Riggleman, Daniel Beck, Eric Nilsson, Millissia Ben Maamar, Michael K. Skinner

**Affiliations:** 1 IVI-RMA València, and IVI Foundation, Health Research Institute La Fe, València, Spain; 2 Dept Ob/Gyn, València University/Instituto de Investigacion Clinica, Hospital Clinico de Valencia (INCLIVA), and Igenomix Foundation, València, Spain; 3 Beth Israel Deaconess Medical Center, Harvard University, Boston, USA; 4 Center for Reproductive Biology, School of Biological Sciences, Washington State University, Pullman, WA 99164-4236 USA

**Keywords:** Autism, Sperm, Epigenetics, Diagnostic, Generational, Father, Offspring

## Abstract

**Background:**

Autism spectrum disorder (ASD) has increased over tenfold over the past several decades and appears predominantly associated with paternal transmission. Although genetics is anticipated to be a component of ASD etiology, environmental epigenetics is now also thought to be an important factor. Epigenetic alterations, such as DNA methylation, have been correlated with ASD. The current study was designed to identify a DNA methylation signature in sperm as a potential biomarker to identify paternal offspring autism susceptibility.

**Methods and results:**

Sperm samples were obtained from fathers that have children with or without autism, and the sperm then assessed for alterations in DNA methylation. A genome-wide analysis (> 90%) for differential DNA methylation regions (DMRs) was used to identify DMRs in the sperm of fathers (*n* = 13) with autistic children in comparison with those (*n* = 13) without ASD children. The 805 DMR genomic features such as chromosomal location, CpG density and length of the DMRs were characterized. Genes associated with the DMRs were identified and found to be linked to previously known ASD genes, as well as other neurobiology-related genes. The potential sperm DMR biomarkers/diagnostic was validated with blinded test sets (*n* = 8–10) of individuals with an approximately 90% accuracy.

**Conclusions:**

Observations demonstrate a highly significant set of 805 DMRs in sperm that can potentially act as a biomarker for paternal offspring autism susceptibility. Ancestral or early-life paternal exposures that alter germline epigenetics are anticipated to be a molecular component of ASD etiology.

## Introduction

Autism spectrum disorder (ASD) is a complex neurological disorder involving deficits in communication, social behaviors and stereotypic movements [1, 2]. The prevalence of ASD in 1975 was reported as 1 in 5000 and then in 2009 as 1 in 110 [3]. The American Centers for Disease Control and Prevention reported a 1 in 88 prevalence in 2012 and then a 1 in 68 in 2014. Although improved diagnosis and current awareness have played a role in this increase, particularly in the first couple decades (1975–2000), the increase in the last two decades is thought to be due to environmental and molecular factors [2–4]. This is supported by twin studies and numerous environmental studies. Genetic studies using genome-wide association studies (GWAS) have identified multiple genetic mutations, but they are correlated with only a small percentage of the autism patients [5]. A recent study identified sperm genetic alterations associated with offspring autism [6]. Combining genetic mutations and altered epigenetics appear to improve associations [7]. Many specific toxicants and factors have been suggested to be involved, but generally more extensive analysis is required [8]. Environmental factors are now believed to be involved in the etiology of autism. A number of molecular alterations in the genome have been correlated to the neurobiology of ASD [2]; however, the specific environmental factors, molecular processes and etiology of autism remain to be fully elucidated.

Although there are both paternal and maternal transmission of ASD, the prevalence of paternal transmission is higher in most populations. One of the main factors proposed to be involved is paternal age [9], with an increased percentage risk of 28% between 40–49 years and nearly 70% when greater than 50 years of age [4]. Increased paternal age has been associated with epigenetic DNA methylation alterations in sperm [10], including specific genes associated with autism [11, 12]. Paternal age-associated DNA methylation alterations have been shown to impact offspring health and disease susceptibility [13, 14]. Therefore, the current study controlled for age at conception and sample collection for the comparison. In addition to paternal age effects, ancestral and early-life exposures to toxicants, abnormal nutrition and stress can also impact sperm DNA methylation to potentially affect disease susceptibility of offspring [15]. The current study was designed to examine the father’s sperm epigenetics (DNA methylation) in families with or without autistic children. The hypothesis examined is that a father’s specific sperm DNA methylation alterations will correlate with offspring autism susceptibility.

Epigenetics is defined as “molecular factors and processes around DNA that regulate genome activity independent of DNA sequence and are mitotically stable.” The molecular factors and processes currently known are DNA methylation, histone modifications, chromatin structural changes, noncoding RNA and RNA methylation [15]. When the epigenetic alterations become programmed in the germ cells (sperm or egg), they have the potential to promote in subsequent generations the epigenetic transgenerational inheritance of disease and phenotypic alterations [15]. Environmental factors that promote these early-life epigenetic alterations have the ability to promote epigenetic inheritance to subsequent generations and dramatically increase disease susceptibility and prevalence [15–17]. The current study was designed to use an epigenome-wide association study approach and develop a potential paternal sperm biomarker for offspring autism susceptibility.

The use of specific sperm epigenetic (DNA methylation) alterations (i.e., biomarkers) could be used for a fathers (i.e., paternal) offspring autism susceptibility, and applications in an assisted reproduction setting could be considered. Although genetic tests are common in assisted reproduction and preimplantation diagnostics, epigenetic analysis is less common. Sperm DNA methylation diagnostics have been proposed for the use in assisted reproduction [18]. The availability of a sperm DNA methylation biomarker for offspring autism susceptibility would allow improved clinical management and early treatment options to be considered. An epigenome-wide association study for DNA methylation alterations in sperm from fathers with or without autistic children was used to identify potential sperm epigenetic alterations as a biomarker for paternal offspring autism susceptibility.

## Results

Paternal males with children affected by autism (case) or without (control) were recruited, and paternal sperm samples were collected at the Andrology Laboratory of IVIRMA Clinic in Valencia, Spain. The sperm sample was collected upon enrollment. Thirty-six patients were enrolled, which included thirteen in the control group, thirteen in the autism case group, and eight or ten for the blinded test groups. The differences (mean ± SD) between the semen analysis for both control and case group are shown in Table 1. Observations from the groups showed no significant difference in age, fathers age at pregnancy, fathers age upon sperm collection, sperm volume, concentration, or sperm concentration between the groups. Progressive sperm motility was greater in the autism case group, with no difference in non-progressive sperm motility, as shown in Table 1a. The motile percentage was higher in the control group, and no difference was observed in the total motile sperm count. One of the control samples, IVI 14, had a very high sperm count of 396.62 million that was outside two standard deviations of the mean (2 ± SD), so the analysis was redone without this sample. When the IVI 14 sample was not used in the analysis, the total sperm number was increased in the autism case group (*p* < 0.02), and the total motile sperm count (time) was increased in the autism case group (*p* < 0.017), as well as the progressive spermatozoa (%) (*p* < 0.019) and immotile % (*p* < 0.019) parameters. In addition to the case and control male age and sperm analysis parameters, Table 1a, all the blinded test set males, Table 1b, c, age and sperm parameters were analyzed and found to also be within the mean ± SD of the case and control samples presented (Additional file 2: Figure S1). Therefore, the blinded test set of individuals was appropriate comparisons with the same clinical parameters.

**Table 1:** Sperm samples and clinical semen analysis.

Study sample						Paternal sperm analysis						
Sample	Age (years)	Father age (years) upon collection	Father age (years) at pregnancy	Collection date of sample	Offspring autism case/control	Volume (mL)	Concentration (mill/mL)	Total of spermatozoa (mill)	Progressive spermatozoa (%)	Non-progressive spermatozoa (%)	Immotile (%)	Total motile sperm count (time)
(a) Sperm samples and clinical analysis												
IVI 1	42	42	31	7/27/15	Case	2.2	83	182.6	58	11	31	105.91
IVI 2	44	45	43	7/28/15	Case	1.5	38	57	42	11	47	23.94
IVI 3	41	41	38	7/29/15	Case	3	68	204	44	11	45	89.76
IVI 4	38	38	34	7/29/15	Case	3.4	66	224.4	35	11	54	78.54
IVI 5	37	37	33	7/30/15	Case	1.4	10	14	50	14	36	7
IVI 6	39	49	40	8/4/15	Case	2.2	23	50.6	31	26	43	15.69
IVI 7	41	41	31	8/12/15	Case	6	47	282	69	14	17	194.58
IVI 8	42	42	32	8/14/15	Case	2.5	91	227.5	60	15	25	136.5
IVI 9	45	45	35	9/9/15	Case	3.5	87	304.5	50	13	37	152.25
IVI 10	–	39	31	9/28/15	Case	3	33.3	99.9	44	9	47	43.96
IVI 11	–	45	39	12/21/15	Case	4	120	480	50	12	38	240
IVI 12	35	37	24	9/5/17	Case	5.4	36.3	196.02	43	3	54	84.29
IVI 13	46	46	35	3/7/16	Case	4.2	17.6	73.92	60	17	23	44.35
IVI 14	40	40	36	10/11/16	Control	2.8	141.65	396.62	55	10	35	218.14
IVI 15	41	41	36	10/11/16	Control	1	34.3	34.3	31	18	51	10.63
IVI 16	46	46	35	10/17/16	Control	2.2	5.5	12.1	29	3	68	3.51
IVI 17	44	44	33	10/20/16	Control	4.8	10.5	50.4	42	12	46	21.17
IVI 18	38	38	31	10/21/16	Control	3.2	1.6	5.12	28	13	59	1.43
IVI 19	36	36	34	11/3/16	Control	6.8	14	95.2	32	3	65	30.46
IVI 20	41	41	41	3/22/17	Control	2.2	91.8	201.96	49	14	37	98.96
IVI 21	42	42	32	5/23/17	Control	1.4	42.1	58.94	37	23	40	21.81
IVI 22	37	37	29	9/6/17	Control	4.2	54	226.8	52	9	39	117.94
IVI 23	43	43	36	9/6/17	Control	1.8	13	23.4	23	20	57	5.38
IVI 24	54	54	27	9/15/17	Control	2.5	52	130	48	7	45	62.4
IVI 25	38	38	34	9/15/17	Control	5.5	1.7	9.35	11	8	81	1.03
IVI 26	43	43	33	9/22/17	Control	1.5	96	144	35	17	48	50.4
Mean ± SD	41 ± 3	42 ± 4	34 ± 5	Offspring autism case		3 ± 1	55 ± 33	184 ± 128	49 ± 11	13 ± 5	38 ± 12	94 ± 71
Mean ± SD	42 ± 5	42 ± 5	34 ± 3	Offspring control		3 ± 2	43 ± 44	107 ± 114	36 ± 13	12 ± 6	52 ± 14	49 ± 63
Statistical comparison	NS	NS	NS	(Case vs. Control), not significant (NS) > 0.05		NS	NS	NS	*p* < 0.01	NS	*p* < 0.01	NS

(b) Blinded test sample set 1 analysis		
BS 1	Identified case	Actual case
BS 2	Identified control	Actual control
BS 3	Identified control	Actual control
BS 4*	Identified control	Actual Case*
BS 5	Identified case	Actual case
BS 6	Identified case	Actual case
BS 7	Identified control	Actual control
BS 8	Identified control	Actual control

(c) IVI blinded test sample set 2 analysis		
BS 9	Identified case	Actual case
BS 10	Identified case	Actual case
BS 11	Identified case	Actual case
BS 12	Identified case	Actual case
BS 13	Identified control	Actual control
BS 14*	Identified control	Actual case*
BS 15	Identified control	Actual control
BS 16	Identified control	Actual control
BS 17	Identified control	Actual control
BS 18	Identified control	Actual control

(a) The samples were provided by IVI-RMA, Valencia, Spain, that collected and performed the clinical analysis of semen volume, sperm concentration and analysis, motility and formal progression analysis, and motility analysis. The mean ± SD for case and control samples is provided. A Students *t* test was used to assess any statistical differences between case and control groups. (b) Blinded test sample set #1 analysis. The analysis was performed to identify the case or control samples and then confirmed once completed after analysis unblinded. (c) Blinded test sample set #2 analysis. IVI-RMA provided an additional ten blinded samples, and following analysis (Identified) was unblinded to assess accuracy (Actual). The false-negative identifications are indicated with an (*) on the labels

The participant demographics and clinical information were similar between the case and control population participants. The ethnicity of all the fathers was Caucasian. No major comorbidities were observed within either the control or case populations. The date of the patient sperm collection, age of the father upon collection and age of the father at conception of child are all not statistically different and provided in Table 1. Although age can impact sperm DNA methylation, the mean age upon sperm collection, which required a 3-year collection period, for the case and control was not statistically different, as given in Table 1. In addition, no statistical difference was observed in the age of the fathers at conception of child, as given in Table 1. All the autistic children were males. Since the focus was on paternal sperm, and due to IRB restrictions, the offspring ASD spectrum severity was not considered. The human subjects approval and informed consent were obtained from all participants prior to the initiation of the study and approved by the Ethics Committee of Valencian Infertility Institute—Reproductive Medicine Associates (IVIRMA) Valencia, Spain, with code, #1311-VLC-136-FC.

**Fig. 1:**
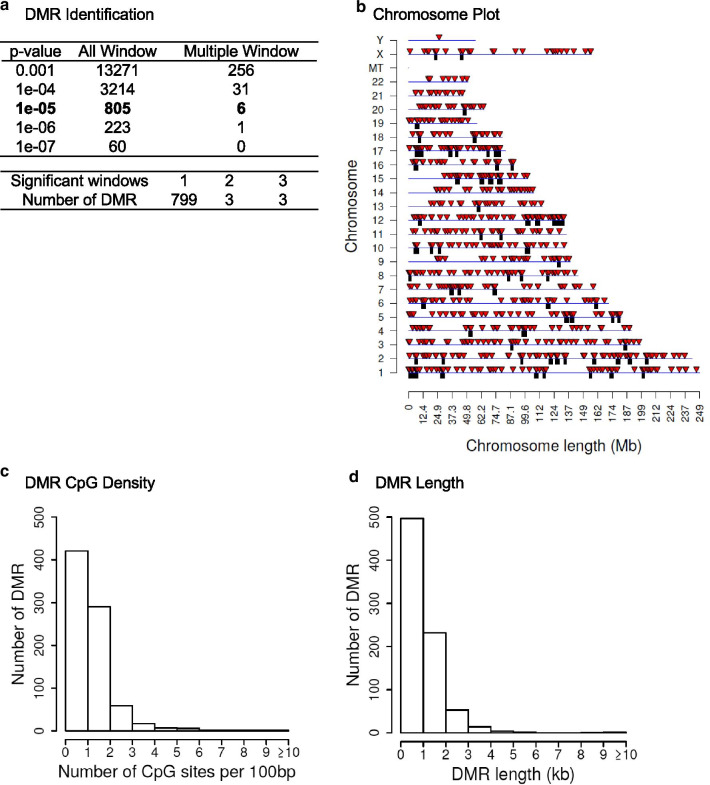
**a** DMR identifications. Autism case versus control sperm DMR analysis. The number of DMRs found using different *p* value cutoff thresholds. The all window column shows all DMRs. The multiple window column shows the number of DMRs containing at least two adjacent significant windows and the number of DMRs with each specific number of significant windows at a *p* value threshold of 1e−05. **b** Autism case versus control patient DMR analysis. The DMR locations on the individual chromosomes are identified. All DMRs at a *p *value threshold of *p* < 1e−05 are shown with the red arrowheads and clusters of DMRs with the black boxes. **c** DMR CpG density in the autism case versus control patient DMRs. The number of DMRs at different CpG densities is indicated. All DMRs at a *p *value threshold of *p* < 1e−05. **d** Autism case versus control patient DMR lengths in kilobases. All DMRs at a *p* value threshold of 1e−05 are shown

Individual patient sperm samples from the collection upon enrollment were prepared for sperm analysis, and an aliquot was taken and flash frozen with liquid nitrogen and stored at − 20 °C until shipment on dry ice for the epigenetic analysis. The samples were thawed, and prior to DNA isolation, the sperm were sonicated to destroy and remove any contaminating somatic cells, as previously described [16]. Due to the sperm nuclei being resistant to sonication, any contaminating somatic cells are removed following sonication. The DNA was extracted from the sperm and then fragmented for a methylated DNA immunoprecipitation (MeDIP) procedure to obtain methylated DNA for analysis to identify differential DNA methylated regions (DMRs). The MeDIP is a genome-wide analysis examining 95% of the genome, which is comprised of low-density CpG regions in comparison with the less than 5% of the genome of high-density CpG regions such as CpG islands. The MeDIP DNA libraries were prepared for next-generation DNA sequencing by creating individual patient sequencing libraries. Samples were then sequenced for bioinformatic analysis, as described in Additional file 1 section. A comparison of the sequences between the control (non-autism children) and case (autism children) participant sperm samples identified DMRs, as shown in Fig. 1a. At a *p* value of *p* < 1e−05 there were 805 DMRs identified with the majority being a single 1-kb window with fewer (i.e., six) having multiple adjacent 1-kb windows involved. The DMRs at a number of *p* values are presented for *p* < 001 to *p* < 1e−07, Fig. 1a. The DMRs at EdgeR *p* < 1e−05 all had false discovery rate (FDR) of *p* < 0.05 and were used for subsequent data analysis. The *p* < 1e−05 was used to optimize DMR numbers and statistical considerations. A list of these DMRs with various genomic features (e.g., CpG density and chromosomal location) are presented in Additional file 3: Table S1. Observations suggest that males with autistic children have a sperm DMR signature that is distinct from males without autistic children (control).

The genomic features of the offspring autism susceptibility DMRs were investigated. The chromosomal locations of the DMRs at *p* < 1e−05 within the human genome are presented in Fig. 1b. The red arrowheads indicate the individual DMRs, and the black boxes represent clusters of DMRs. The DMRs are present on all chromosomes. The CpG density of the DMRs is generally less than 10 CpG per 100 bp with 1–3 CpG predominant for the paternal offspring autism susceptibility DMRs, as shown in Fig. 1c. The size of the DMRs was predominantly 1–3 kb for the sperm DMRs, as shown in Fig. 1d. Additional genomic features are presented in Additional file 4: Table S1. The log-fold change (LFC) in DMA methylation in Additional file 3: Table S1 demonstrated for the 805 DMR in the autism group that 303 have an increase in DNA methylation and 502 have a decrease in DNA methylation. The autism DMRs involved a 38% increase or 62% decrease in DNA methylation. Therefore, the majority of the sperm DMRs had low CpG density, termed a CpG desert, and were 1 kb in length with either an increase or decrease in DNA methylation.

**Fig. 2:**
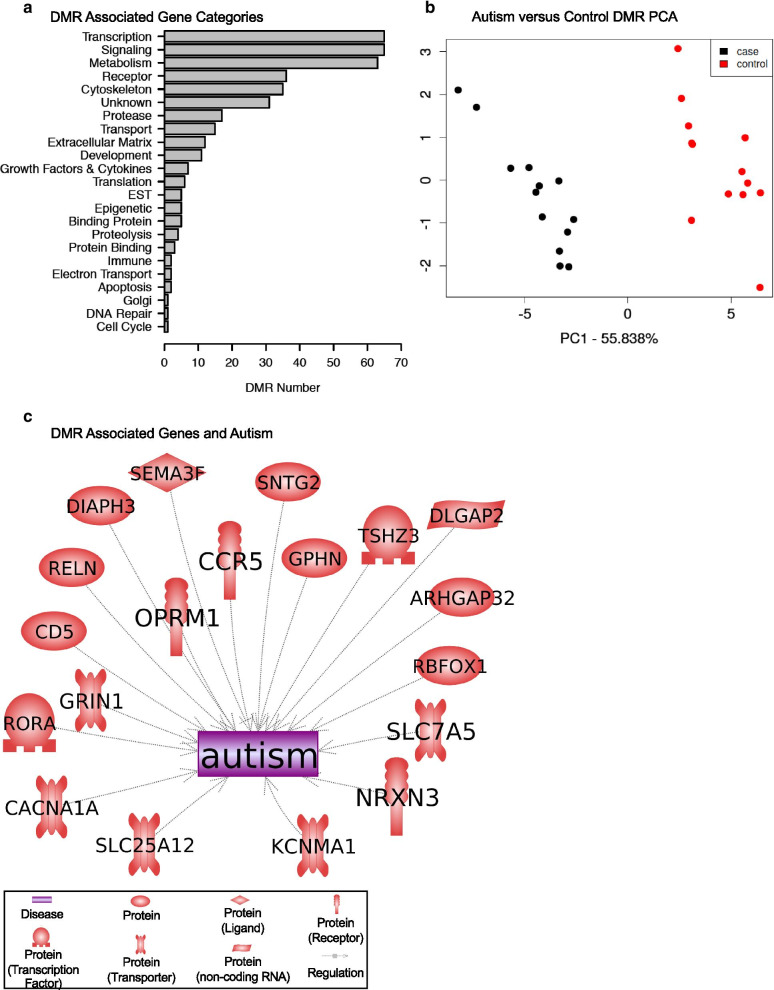
**a** DMR-associated gene categories. DMRs at a *p* value threshold *p* < 1e−05 are shown. **b** Autism case versus control DMR PCA. PCA for DMRs at *p* < 1e−05. The first two principal components used and samples color code index indicated. The underlying data are the RPKM read depth for all DMRs. **c** DMR-associated genes and autism. The paternal offspring autism-susceptible DMRs previously shown to correlate with autism and associated neurodegenerative disease are presented. DMR-associated genes from the current study were compared to genes associated with autism in the published literature using Pathway Studio software (Elsevier, Inc.). Those that were in common are depicted

The paternal offspring autism susceptibility sperm DMR-associated genes and corresponding gene functional categories were determined, as presented in Additional file 4: Table S1. The total autism 805 DMRs had 193 with no DMR gene associations (24%), and the DMRs were intergenic and not associated with genes. From the 612 DMR with gene associations (76%), there were 493 DMR that overlapped with annotated genes. There were 17 DMR in the 1–1000 bp and 62 DMR in the 1–5 kb proximal promoter regions. There were 40 DMRs in the 5–10 kb distal promoter region. Therefore, approximately 20% of the DMR are in the proximal and distal promoter region and 80% overlapping the gene annotation regions. There were 193 DMRs that were intergenic. These DMRs are intergenic and not proximal to genes, but can influence gene expression events for megabase distances through ncRNA and chromatin structure alterations, as previously described [19, 20]. Genes within 10 kb of a DMR were identified, which has previously been shown to be optimal for both proximal and distal promoter regions and epigenetic associations [21]. The functional categories corresponding to each DMR-associated gene are summarized in Fig. 2a. The signaling, transcription and metabolism functional categories are predominant. This reflects that these gene functional categories have the highest number of genes within them. A comparison of previously identified genes associated with neurodegeneration, neurodevelopment and autism with the DMR-associated genes of this study is summarized in Fig. 2b. These autism-associated genes have previously been shown to be regulated or involve genetic mutations within autism patients, and the gene symbols, descriptions and associated references are presented in Additional file 5: Table S2. The DMR-associated genes were also used in a gene pathway or gene set analysis to identify associated pathways. Interestingly, the top pathway or gene set identified was autism, and the majority of the subsequent pathways with greater than three genes were all neurodevelopmental- or neurobiology-associated pathways, as given in Table 2. All those gene sets were found to be significant, and a list of the specific DMR-associated genes is provided, as given in Table 2. As shown with all the DMR-associated genes, Additional file 5: Table S2, the associated genes in Table 2 also had approximately a 50% mixture of genes with an increase or decrease in DNA methylation. Therefore, the DMR-associated genes did correlate well with previously identified autism- and neurodevelopment-associated genes. Since the sperm DMRs will impact the embryo epigenomes and transcriptomes of all subsequent somatic cells, this dynamic cascade of developmental epigenetics needs to be considered in potential links in sperm epigenetics and potential neurological impacts on autism.

**Table 2:** DMR-associated gene pathways or gene sets.

Pathway or gene set name	Total # of neighbors	Pathology gene set pathway	Overlap	Percent overlap	Overlapping genes	*p *value
Protein regulators of autism	313	Autism	19	6	RBFOX1, CD5, CCR5, GRIN1, GPHN, SLC7A5, SNTG2, RELN, ARHGAP32, OPRM1, DLGAP2, DIAPH3, CACNA1A, KCNMA1, TSHZ3, SLC25A12, NRXN3, SEMA3F, RORA	9.02E−05
Protein regulators of intellectual disability	616	Intellectual disability	28	4	SRGAP3, ERBB4, DMD, TG, AHI1, ANKRD11, CHL1, ST3GAL5, BCKDK, CDK19, DLGAP2, NARS2, PHF21A, KDM2B, CAMTA1, MCPH1, CREBBP, ARHGEF10, TCF4, LIMK1, STIM1, RELN, PRMT7, DPYSL2, SORL1, LRMDA, CDH18, EXOC4	3.42E−04
Protein regulators of neurodevelopmental disorder	273	Neurodevelopmental disorder	16	5	RBFOX1, SRGAP3, GRIN1, LIMK1, AHI1, DOCK3, ANKS1B, ELP4, ANKRD11, RELN, DPYSL2, ST3GAL5, AGAP1, MCPH1, CREBBP, TCF4	4.70E−04
Protein regulators of psychiatric disorder	538	Psychiatric disorder	25	4	GRIN2B, PDE2A, ERBB4, AHI1, CCKBR, ANKS1B, DLGAP1, CCDC141, OPRM1, PDLIM5, GABBR1, SORCS2, CREBBP, ARHGEF10, TCF4, DNMT1, RBFOX1, GPHN, USP46, RELN, DPYSL2, ADRA2C, IMMP2L, NRXN3, ALK	5.12E−04
Protein regulators of intellectual impairment	18	Intellectual impairment	4	21	GRIN2B, GRIN1, DMD, RELN	5.95E−04
Protein regulators of estrogen receptor-positive breast cancer	86	Estrogen receptor-positive breast cancer	8	9	KDM6B, PPARG, ERBB4, PIK3CD, NDRG1, CREBBP, SLC7A5, STIM1	6.90E−04
Protein regulators of behavioral disorder	147	Behavioral disorder	10	6	GRIN2B, GRIN1, ERBB4, PARK7, STX1A, RORA, DNMT1, TMEM173, AP2B1, OPRM1	1.80E−03
Protein regulators of severe visual impairment	14	Severe visual impairment	3	20	PAX2, AIPL1, MERTK	3.39E−03

The pathway total number of neighbors for genes, pathology gene set pathway, overlapping genes, percent overlap, overlapping gene symbols and statistical *p *value are presented

The final analysis examined the statistical significance and validation of the DMRs identified. A permutation analysis was performed on the DMRs to determine whether the DMRs were due to background variation in the data or randomly generated. The permutation analysis shows the number of DMRs generated from the control versus autism case comparison was significantly greater than the DMRs generated from random subset analysis, Additional file 3: Figure S2. The red line to the right indicates the comparison DMRs versus the low numbers from the random subset comparison. Another analysis involved a cross-validation of the DMRs and demonstrated approximately 80% accuracy in the confirmation of the DMRs to assess autism susceptibility [22]. A principal component analysis (PCA) of the control male sperm without an autistic child versus the male sperm with an autistic child is presented in Fig. 2c. A clear separation of the DMR principal components is seen between the groups. This demonstrates a distinction between the DMR principal components.

The final validation involved using blinded test sets of samples for analysis to identify and assess the accuracy to determine actual case or control samples. Three different molecular analyses of the original 13 cases and 13 controls were performed and combined for this test set analysis. The test set analysis involved four independent analyses that were combined for the final analysis. The first test set involved eight blinded samples selected from the main analysis samples and reanalyzed (BS1–BS8) and identified as actual case or control sample, as given in Table 1b. All were accurately identified, except one false negative which was identified as a control, but actually was a case sample. A second set of ten blinded test samples (BS9–BS18) was provided by IVI-RMA clinical collaborators and was also identified in an independent analysis as case or control, as given in Table 1c. All were accurately identified, except one false negative which was identified as a control, but was actually a case sample. Therefore, the blinded test set analysis indicated all but one in each test set were accurately identified for approximately a 90% accuracy in the analysis. Since multiple analysis was used for this blinded test set analysis, random batch effect outlier DMRs identified were removed to optimize the analysis. Although significantly more validation with larger clinical test sets is needed, the current study provides the proof of concept that epigenetic biomarkers potentially exist and may be used to diagnose that a father may potentially have a child with a susceptibility for autism.

## Discussion

The frequency of autism in the population has dramatically increased over tenfold the past several decades. This increase appears to be due in part to increased diagnosis efficiency from 1975 to the early 2000s, as well as greater public awareness of the disease [3]. The more recent increase in the last couple of decades suggests environmental factors, and exposures also have a critical role in autism prevalence. Although many suggestions have been made on specific toxicants and factors being involved, more extensive analysis and better understanding of autism etiology are needed to understand this increase in autism frequency [8]. An example is the suggestion-assisted reproduction and in vitro fertilization is involved, but follow-up studies demonstrated no risk of ASD in children born after assisted reproduction [23, 24]. One factor that has been correlated with autism is paternal age [4, 9, 15] and sperm DNA methylation alterations [11, 25]. Previous studies have shown a hypermethylation of sperm DNA is associated with male infertility, abnormal sperm parameters and increasing age [13, 26, 27]. Therefore, the majority of DMR involve an increase in DNA methylation when associated with infertility or age. The current study demonstrated 60% of the DMRs have a decrease in DNA methylation, and 40% of DMRs an increase in DNA methylation, Additional file 4: Table S1. Therefore, a mixture of an increase and decrease in methylation is observed, which is distinct from the sperm hypermethylation observed in male infertility and aging [13, 26, 27]. Since all the paternal subjects were similar in age and fertile (Table 1), the current study observations appear to be distinct from infertility and aging-related DNA hypermethylation. Although some participants from both control and case populations were involved in in vitro fertilization upon sperm collection, male factor infertility was not involved. No differences in demographics or clinical variables were observed. The age upon sperm collection between the case and control was not statistically different, as given in Table 1. In addition, the age at conception of child was also not statistically different between case and control participants. The comparison was biased on age of sperm collection to control for age differences to minimize DNA methylation variation. However, similar observations were also obtained considering age of conception of child. Therefore, the current study was designed to identify sperm epigenetic alterations (i.e., biomarkers) to assess a father’s potential ability to transmit autism susceptibility to his offspring.

Altered germline epigenetics has been shown to impact offspring health later in life, and if permanently programmed, to promote the epigenetic transgenerational inheritance of disease and pathology to subsequent generations [15, 16]. Since sperm or egg epigenetics can impact the zygote epigenetics and transcription following fertilization, as well as the subsequent stem cell population in the early embryo epigenome and transcriptome, all subsequently derived somatic cells also have the potential to have an altered cell-type specific epigenomes and transcriptomes later in development [15, 28]. This molecular alteration has been shown to be associated with adult somatic cell epigenetics, transcriptomes and associated diseases [29–31]. The ability of an ancestral or early-life exposure to impact the germline epigenetics to subsequently impact the offspring epigenetics and susceptibility to develop pathology and disease has been established [15–17, 28–31] and is anticipated to be a component of autism etiology as well. The current study observations support the concept that similar events may contribute to autism etiology.

The application of a sperm molecular diagnostic is optimally used in an assisted reproduction setting. Routine semen analysis and genetic testing are used in most in vitro fertilization clinical settings. Although epigenetic analysis is not as routine, the proposal for such analysis has been made [18]. The analysis of male infertility using sperm DNA methylation alterations has been developed [32]. Epigenetic alterations (DNA methylation) in sperm have been shown to associate in fathers of families with autistic children [33]. That study used a targeted array-based approach that focused on high-density CpG islands that constitutes approximately 1% of the genome, but does demonstrate such an analysis is feasible. The current study was designed to use a genome-wide approach to identify altered DNA methylation for paternal sperm and offspring autism susceptibility.

Although genetics will be involved in autism etiology, genome-wide association studies (GWAS) have demonstrated generally less than 1% of the patients with a specific disease, such as neurodegenerative disease that has a correlated genetic mutation [34]. ASD is similar to only a few percent correlation with associated genetic mutations [35]. An additional molecular mechanism to consider for ASD disease etiology involves epigenetics. The current study uses a more epigenome-wide association study approach to investigate sperm DNA methylation in fathers with or without autistic children. A procedure to assess DNA methylation alterations in low-density CpG regions, that constitute over 95% of the human genome, was used in comparison with the high-density CpG procedures previously used. A significant signature of differential DNA methylation regions (DMRs) was identified comparing the sperm from fathers with or without autistic children. The genomic features of the DMRs were identified and demonstrated generally 1-kb lengths and low-density CpG regions. The DMR-associated genes were identified, and a number of previously identified autism-linked genes were present (Fig. 2b, Additional file 5: Table S2 and Table 2). In regard to the autism sperm DMR biomarkers, a separation in a principal component analysis (PCA) was observed. In addition to this validation, the permutation and cross-validation analyses help demonstrate the robustness and sensitivity of the analysis. The validation studies with blinded sample sets accurately identified the majority of case and control samples, but potential false-negative identification of case samples was observed. The observations demonstrate the paternal sperm epigenetic analysis is potentially effective at identifying offspring susceptibility for autism, but the current analysis needs to be improved with expanded clinical trials.

Although an epigenetic signature was identified for paternal transmission of susceptibility of autism children, which was identified and statistically significant, a limitation of the current study is the low number of samples used for the analysis. Although epigenetic alterations occur at a significantly higher frequency than genetics, expanded clinical trials are required with increased numbers, greater ethnic diversity, and more thorough assessment of the impacts of paternal age. The impacts of these variables need to be elucidated to improve and expand the accuracy of the analysis. The expanded clinical trial with greater numbers and diverse subpopulations is essential to develop a useful diagnostic. However, the current study does provide the proof of concept; such a diagnostic can be developed.

Applications of the paternal offspring autism susceptibility biomarker/diagnostic will potentially improve the health care for ASD patients. This would allow IVF patients to assess risk and determine management procedures. Importantly, this would allow clinicians to plan the offspring’s clinical management options more efficiently. Potential preventative treatments could be considered to reduce the severity of the autism spectrum disorder. The availability of the assay could also be used in a research setting to facilitate the identification of environmental factors potentially involved in the ASD etiology. Therefore, potential therapeutic and preventative options not previously considered could be taken.

The current study identified a genome-wide signature of DNA methylation sites that are associated with the paternal transmission of offspring autism susceptibility. Although a large clinical trial is needed to further validate the biomarkers and potential diagnostic, the current study provides the proof of concept for the assay and biomarkers. Therefore, the identification of offspring susceptibility can be assessed, allowing better clinical management of ASD. The potential for therapy options can be expanded to improve health care for ASD. Such epigenetic biomarkers are anticipated to exist for many disease and pathology conditions, which will facilitate the future preventative medicine strategies for health care. In addition, the current study suggests epigenetic inheritance may play a role in ASD etiology and explain the paternal transmission prevalence of the disease.

## Methods summary

### Clinical sample collection

A single-center (IVIRMA Valencia, Spain) prospective and open clinical study was performed. The participant approval and informed consent were obtained from all participants prior to the clinical sample collection. The study protocols were approved by the Institutional Review Board Ethics Committee of Valencian Infertility Institute—Reproductive Medicine Associates (IVIRMA) Valencia, Spain, with code, #1311-VLC-136-FC. All research was performed in accordance with relevant guidelines/regulations. The study was not designed for, nor did the IRB involve, the ability to correlate autism child clinical information to be correlated. The semen was analyzed as described in Additional file 1. Samples were immersed in liquid nitrogen and then stored at −20 °C prior to analysis.

### Epigenetic analysis, statistics and bioinformatics

Somatic cell contamination was removed by sonication, and the sperm DNA was isolated as previously described [16]. Methylated DNA immunoprecipitation (MeDIP), followed by next-generation sequencing (MeDIP-Seq), was performed. MeDIP-Seq, sequencing libraries, next-generation sequencing, and bioinformatics analysis were performed as described [16] and are found in Additional file 1. The statistical analysis and validation protocols were performed as previously described [16] and are found in Additional file 1. All molecular data has been deposited into the public database at NCBI (GEO # GSE157417), and R code computational tools are available at GitHub (https://github.com/skinnerlab/MeDIP-seq) and www.skinner.wsu.edu.

## Supplementary Information

**Additional file 1:** Supplemental methods.

**Additional file 2: Figure S1**. Clinical group statistic comparison. The various sperm/semen characteristics in the Study case and control group were compared with the Blind group. The n-value, mean, standard deviation, and standard error mean are presented. The blind groups are within the mean ± SD of the case and control study.

**Additional file 3: Figure S2**. Permutation analysis. The number of DMR for autism case versus control patient comparison for all permutation analyses. The vertical red line shows the number of DMR found in the original analysis. All DMRs are defined using an edgeR *p* value threshold of *p* < 1e−05.

**Additional file 4: Table S1**. DMR lists at *p* < 1e−05 with presentation of name, chromosomal location, DMR start and stop nucleotide number for chromosome, length (bp), number of 1 kb significant windows, minimum *p* value, CpG number and density, DMR maximum, log-fold change (maxLFC) (+ increase DNA methylation and − decrease DNA methylation), and gene association within 10 kb and gene functional categories.

**Additional file 5: Table S2**. Gene and protein regulators of autism. The DMR-associated autism-related genes with symbol, description, and relevant references PubMed ID (PMID) numbers.

## Data Availability

All molecular data have been deposited into the public database at NCBI (GEO # GSE157417), and R code computational tools are available at GitHub (https://github.com/skinnerlab/MeDIP-seq) and www.skinner.wsu.edu.

## Abbreviations

ASD: Autism spectrum disorder
IVF: In vitro fertilization
DMRs: Differential DNA methylation regions
GWAS: Genome-wide association studies
MeDIP: Methylated DNA immunoprecipitation
MeDIP-Seq: Methylated DNA immunoprecipitation followed by next-generation sequencing
FDR: False discovery rate
LFC: Log-fold change
PCA: Principal component analysis
BS: Blinded samples
