# Supplementary material for: Sperm DNA methylation epimutation biomarker for paternal offspring autism susceptibility
Source: Clin Epigenetics. 2021 Jan 7;13:6. doi: 10.1186/s13148-020-00995-2 (PMC7789568; doi:10.1186/s13148-020-00995-2)
Supplement: Supplementary file 1 — Additional file 1: Supplemental methods. [file 13148_2020_995_MOESM1_ESM.pdf]

Sperm DNA Methylation Epimutation Biomarker for Paternal Offspring Autism Susceptibility

Nicolás Garrido[1], Fabio Cruz[1], Rocio Rivera Egea[1], Carlos Simon[2], Ingrid Sadler-Riggleman[3], Daniel Beck[3], Eric Nilsson[3], Millissia Ben Maamar[3], and Michael K. Skinner[3]*

[1]IVI-RMA València, and IVI Foundation, Health Research Institute La Fe, València, Spain

[2] Dept Ob/Gyn, València University/Instituto de Investigacion Clinica, Hospital Clinico de Valencia (INCLIVA), and Igenomix Foundation, València, Spain, and Beth Israel Deaconess Medical Center, Harvard University, Boston, MA, USA

[3]Center for Reproductive Biology
School of Biological Sciences
Washington State University
Pullman, WA, 99164-4236, USA

SUPPLEMENTARY MATERIAL

SUPPLEMENTAL METHODS

Clinical sample collection and analysis

A single center (IVIRMA Valencia) performed the prospective and open clinical study. The participant approval and informed consent was obtained from all participants prior to the clinical sample collection. The IRB study was approved by the Ethics Committee of Valencian Infertility Institute - Reproductive Medicine Associates (IVIRMA) Valencia, Spain, with code, #1311-VLC-136-FC. All research was performed in accordance with relevant guidelines/regulations. We included two groups (sperm from father with offspring with autism (case) or without (control)). The men included caucasians between 26 and 54 years of age with a total sperm concentration (concentration in millions/mL x volume in mL). Sperm samples were frozen with liquid nitrogen and stored (-20 C) for the subsequent epigenetic analysis.

DNA Preparation –

Frozen human sperm samples were stored at -20 C and thawed for analysis. Prior to DNA analysis, the contaminating somatic cells were destroyed and removed, due to sperm nuclei resistance to sonication. Genomic DNA from sperm was prepared as follows: A 400 µl of sperm suspension was used and centrifuged for 10 minutes at low speed to concentrate sperm. The supernatant was discarded, and the pellet resuspended in 100 ul PBS. Then 820 µl DNA extraction buffer (50 mM Tris pH 8, 10 mM EDTA pH 8, 0.5% SDS) and 80 µl 0.1 M Dithiothreitol (DTT) were added and the sample incubated at 65 C for 15 min. Proteinase K (20 mg/ml) (80 µl) was added, and the sample was incubated on a rotator at 55 C for at least 2 hours. After incubation, 300 µl of protein precipitation

solution (Promega, A795A, Madison, WI) was added, the sample was mixed and incubated on ice for 15-30 min, then spun at 4 C at 13,000 rpm for 30 min. The supernatant was transferred to a fresh tube, then precipitated over night at -20 C with the same volume. 100% isopropanol and 2 µl glycoblue. The sample was then centrifuged, and the pellet was washed with 75% ethanol, then air-dried and resuspended in 100 µl H2O. DNA concentration was measured using the Nanodrop (Thermo Fisher, Waltham, MA). The freeze-thaw will destroy any contaminating somatic cells in the sperm collection.

Methylated DNA Immunoprecipitation (MeDIP) –

Methylated DNA Immunoprecipitation (MeDIP) with genomic DNA was performed as follows: individual sperm DNA samples (2-4 ug of total DNA) were diluted to 130 µl with 1x Tris-EDTA (TE, 10 mM Tris, 1 mM EDTA) and sonicated with the Covaris M220 using the 300 bp setting. Fragment size was verified on a 2% E-gel agarose gel. The sonicated DNA was transferred from the Covaris tube to a 1.7 ml microfuge tube, and the volume was measured. The sonicated DNA was then diluted with TE buffer (10mM Tris HCl, pH7.5; 1mM EDTA) to 400 µl, heat-denatured for 10 min at 95 C, then immediately cooled on ice for 10 min. Then 100 µl of 5X IP buffer and 5 µg of antibody (monoclonal mouse anti 5-methyl cytidine; Diagenode #C15200006) were added to the denatured sonicated DNA. The DNA-antibody mixture was incubated overnight on a rotator at 4 C. The following day magnetic beads (Dynabeads M-280 Sheep anti-Mouse IgG; 11201D) were pre-washed as follows: The beads were resuspended in the vial, then the appropriate volume (50 µl per sample) was transferred to a microfuge tube. The same volume of Washing Buffer (at least 1 mL 1XPBS with 0.1% BSA and 2mM EDTA) was added and

the bead sample was resuspended. The tube was then placed into a magnetic rack for 1-2 min and the supernatant was discarded. The tube was removed from the magnetic rack and the beads were washed once. The washed beads were resuspended in the same volume of 1xIP buffer (50 mM sodium phosphate ph7.0, 700 mM NaCl, 0.25% TritonX-100) as the initial volume of beads. 50µl of beads were added to the 500µl of DNA-antibody mixture from the overnight incubation, then incubated for 2 hours on a rotator at 4 C. After the incubation, the bead-antibody-DNA complex was washed three times with 1X IP buffer as follows: The tube was placed into a magnetic rack for 1-2 min and the supernatant was discarded, then the magnetic bead antibody pellet was washed with 1xIP buffer 3 times. The washed bead antibody DNA pellet was then resuspended in 250 µl digestion buffer with 3.5 µl Proteinase K (20mg/ml). The sample was incubated for 2-3 hours on a rotator at 55 C, then 250 µl of buffered Phenol-Chloroform- Isoamylalcohol solution was added to the sample, and the tube was vortexed for 30 sec and then centrifuged at 14,000rpm for 5 min at room temperature. The aqueous supernatant was carefully removed and transferred to a fresh microfuge tube. Then 250 µl chloroform were added to the supernatant from the previous step, vortexed for 30 sec and centrifuged at 14,000rpm for 5 min at room temperature. The aqueous supernatant was removed and transferred to a fresh microfuge tube. To the supernatant 2µl of glycoblue (20mg/ml), 20µl of 5M NaCl and 500µl ethanol were added and mixed well, then precipitated in -20 C freezer for 1 hour to overnight. The precipitate was centrifuged at 14,000rpm for 20 min at 4 C and the supernatant was removed, while not disturbing the pellet. The pellet was washed with 500µl cold 70% ethanol in -20 C freezer for 15 min then centrifuged again at 14,000rpm for 5 min at 4 C and the supernatant was discarded. The tube was spun again

briefly to collect residual ethanol to the bottom of the tube and as much liquid as possible was removed with gel loading tip. The pellet was air-dried at RT until it looked dry (about 5 min) then resuspended in 20μl H2O or TE. DNA concentration was measured in Qubit (Life Technologies) with ssDNA kit (Molecular Probes Q10212).

***MeDIP-Seq Analysis* –**

The MeDIP DNA samples (50 ng of each) were used to create libraries for next generation sequencing (NGS) using the NEBNext Ultra RNA Library Prep Kit for Illumina (San Diego, CA) starting at step 1.4 of the manufacturer's protocol to generate double stranded DNA. After this step the manufacturer's protocol was followed. Each sample received a separate index primer. NGS was performed at WSU Spokane Genomics Core using the Illumina HiSeq 2500 with a PE50 application, with a read size of approximately 50bp and approximately 20-50 million reads per sample, and 6-7 sample libraries each were run in one lane.

Molecular Bioinformatics and Statistics –

Basic read quality was verified using information produced by the FastQC program (1). Reads were filtered and trimmed to remove low quality base pairs using Trimmomatic (2). The reads for each sample were mapped to the GRCh38 human genome using Bowtie2 (3) with default parameter options. The mapped read files were then converted to sorted BAM files using SAMtools (4). To identify DMR, the reference genome was broken into 1000 bp windows. The MEDIPS R package (5) was used to calculate differential coverage between control and exposure sample groups. The edgeR p-value (6) was used to determine the relative difference between the two groups for each

genomic window. Windows with an edgeR p-value less than 10^{-5} were considered DMRs. The DMR edges were extended until no genomic window with an edgeR p-value less than 0.1 remained within 1000 bp of the DMR. CpG density and other information was then calculated for the DMR based on the reference genome. DMR were annotated using the biomaRt R package (7) to access the Ensembl database (8). The genes that overlapped with DMR were then input into the KEGG pathway search (9, 10) to identify associated pathways. The DMR associated genes were then sorted into functional groups using information provided by the DAVID (11) and Panther (12) databases incorporated into an internal curated database (www.skinner.wsu.edu under genomic data). All MeDIP-Seq genomic data obtained in the current study have been deposited in the NCBI public GEO database (GEO #: GSE157417).

A permutation analysis to determine the significance of the number of DMR identified for each comparison was performed. For this analysis, samples from the two treatment groups were randomly assigned group membership. The number of samples in each treatment group was held constant. Twenty random permutations of each analysis were performed to obtain a null distribution for the expected number of DMR. In addition, a leave one out cross-validation analysis was performed to estimate the performance of the DMR set at predicting the classification of an unknown sample. For this analysis, each sample was systematically removed from the analysis. The DMR resulting from the cross-validation analysis were then used as input data for predicting the class of the remaining sample. The prediction was done using linear discriminant analysis.

Blinded test set analysis was performed to classify test samples into case or control groups. The first blinded test set (BS1-8) were blinded from the original sample

set and reanalyzed, and the second blinded test set (BS9-18) were new samples provided by IVI-RNA Valencia. For analysis, the two additional blinded test sets of samples were processed and sequenced with independent MeDIP procedures. The additional blinded sample set analyses were analyzed with additional analysis of the original case and control samples. The original case and control analysis used three separate repeat MeDIP sequencing analyses, and blinded test sets four separate repeat MeDIP sequencing analyses, that were combined to reduce batch effects and remove outlier samples to optimize the analysis. The random outliers (IVI 1-8) batch effects that developed in the multiple analysis were removed to optimize the blinded test set analysis. The multiple analysis data sets were combined from each replicate analysis and used for the final analysis of case and control. Equal reads from each of the replicates for each sample were merged into a single combined group for final case or control classification. The PCA and cluster dendrogram analyses were used for case or control identification.

Clinical Sample Statistical analysis

In order to characterize clinical parameters of both groups (control and case group), a numerical descriptive analysis has been made using the mean with standard deviation (SD) and the median (1st and 3rd quartile). The baseline differences between the case group and the control group were then compared in all variables analyzed. For this analysis, mixed linear regression models for several measures per patient (semen volume and sperm concentration) were used, and in the case of motility a beta logistic regression model was performed given its percentage character. The mixed models control the non-independence of data given that there are several measures per patient. The statistical

analyses were performed with the statistical software R (version 3.4.1) and the packages nlme (version 3.1-131), lme4 (1.1-13), glmmADMB (0.8.3.3) and betareg (version 3.1-0). A p-value of less than 0.05 was considered statistically significant.

SUPPLEMENTAL REFERENCES

1. Andrews S. FastQC: a quality control tool for high throughput sequence data. UK2010 [Available from: https://www.bioinformatics.babraham.ac.uk/projects/fastqc/.

2. Bolger AM, Lohse M, Usadel B. Trimmomatic: a flexible trimmer for Illumina sequence data. Bioinformatics. 2014;30(15):2114-20.

3. Langmead B, Salzberg SL. Fast gapped-read alignment with Bowtie 2. Nature methods. 2012;9(4):357-9.

4. Li H, Handsaker B, Wysoker A, Fennell T, Ruan J, Homer N, et al. The Sequence Alignment/Map format and SAMtools. Bioinformatics. 2009;25(16):2078-9.

5. Lienhard M, Grimm C, Morkel M, Herwig R, Chavez L. MEDIPS: genome-wide differential coverage analysis of sequencing data derived from DNA enrichment experiments. Bioinformatics. 2014;30(2):284-6.

6. Robinson MD, McCarthy DJ, Smyth GK. edgeR: a Bioconductor package for differential expression analysis of digital gene expression data. Bioinformatics. 2010;26(1):139-40.

7. Durinck S, Spellman PT, Birney E, Huber W. Mapping identifiers for the integration of genomic datasets with the R/Bioconductor package biomaRt. Nature protocols. 2009;4(8):1184-91.

8. Cunningham F, Amode MR, Barrell D, Beal K, Billis K, Brent S, et al. Ensembl 2015. Nucleic acids research. 2015;43(Database issue):D662-9.

9. Kanehisa M, Goto S. KEGG: kyoto encyclopedia of genes and genomes. Nucleic acids research. 2000;28(1):27-30.

10. Kanehisa M, Goto S, Sato Y, Kawashima M, Furumichi M, Tanabe M. Data, information, knowledge and principle: back to metabolism in KEGG. Nucleic acids research. 2014;42(Database issue):D199-205.

11. Huang da W, Sherman BT, Lempicki RA. Systematic and integrative analysis of large gene lists using DAVID bioinformatics resources. Nature protocols. 2009;4(1):44-57.

12. Mi H, Muruganujan A, Casagrande JT, Thomas PD. Large-scale gene function analysis with the PANTHER classification system. Nature protocols. 2013;8(8):1551-66.
