# Supplementary material for: Sperm DNA methylation epimutation biomarker for paternal offspring autism susceptibility
Source: Clin Epigenetics. 2021 Jan 7;13:6. doi: 10.1186/s13148-020-00995-2 (PMC7789568; doi:10.1186/s13148-020-00995-2)
Supplement: Supplementary file 2 — Additional file 2: Figure S1. Clinical group statistic comparison. The various sperm/semen characteristics in the Study case and control group were compared with the Blind group. The n-value, mean, standard deviation, and standard error mean are presented. The blind groups are within the mean ± SD of the case and control study. [file 13148_2020_995_MOESM2_ESM.pdf]

Supplemental Figure S1

Clinical Group Statistic Comparisons

| | Group | N | Mean | Std. Deviation | Std. Error Mean |
|---|---|---|---|---|---|
| Volume | STUDY | 26 | 3,162 | 1,5557 | ,3051 |
| | BLIND | 18 | 3,561 | 1,5473 | ,3647 |
| Density | STUDY | 26 | 49,1673 | 38,50825 | 7,55209 |
| | BLIND | 18 | 63,4694 | 37,45816 | 8,82897 |
| Total Sperm Count | STUDY | 26 | 145,5627 | 125,36631 | 24,58636 |
| | BLIND | 18 | 196,5394 | 118,34073 | 27,89318 |
| Progressive | STUDY | 26 | 42,615 | 13,1608 | 2,5810 |
| | BLIND | 18 | 46,222 | 13,2557 | 3,1244 |
| Nonprogressive | STUDY | 26 | 12,462 | 5,6372 | 1,1056 |
| | BLIND | 18 | 11,500 | 4,9259 | 1,1611 |
| Immotile | STUDY | 26 | 44,923 | 14,2378 | 2,7923 |
| | BLIND | 18 | 42,278 | 15,2231 | 3,5881 |
| Total Motile Count | STUDY | 26 | 71,5396 | 69,64298 | 13,65811 |
| | BLIND | 18 | 98,4044 | 67,97268 | 16,02131 |
