# Supplementary figures and images for: Sperm DNA methylation epimutation biomarker for paternal offspring autism susceptibility
Source: Clin Epigenetics. 2021 Jan 7;13:6. doi: 10.1186/s13148-020-00995-2 (PMC7789568; doi:10.1186/s13148-020-00995-2)

Permutation Analysis

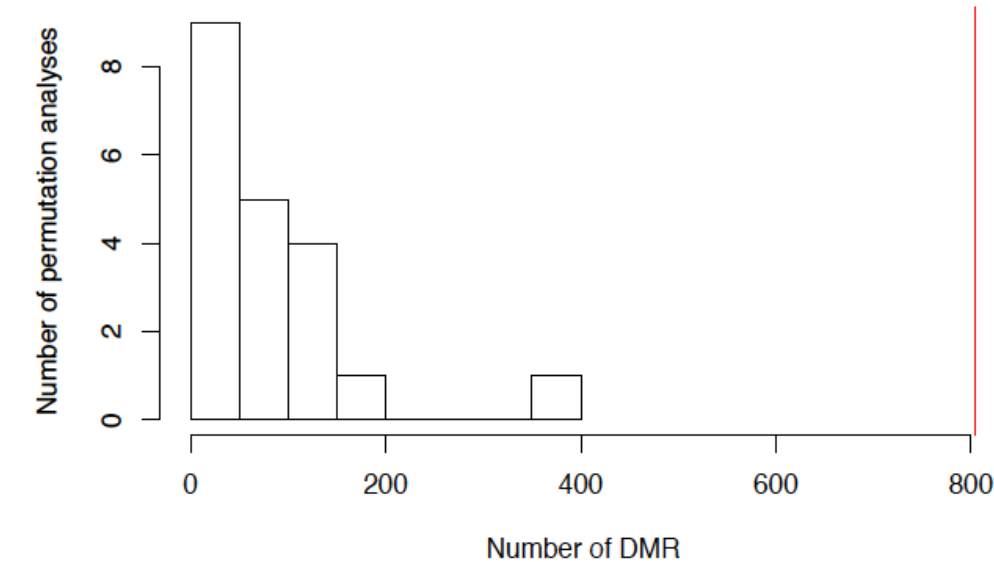

Supplement: Supplementary file 3 — Additional file 3: Figure S2. Permutation analysis. The number of DMR for autism case versus control patient comparison for all permutation analyses. The vertical red line shows the number of DMR found in the original analysis. All DMRs are defined using an edgeR p value threshold of p < 1e−05. [file 13148_2020_995_MOESM3_ESM.pdf]
