# Supplementary material for: Sperm DNA methylation epimutation biomarker for paternal offspring autism susceptibility
Source: Clin Epigenetics. 2021 Jan 7;13:6. doi: 10.1186/s13148-020-00995-2 (PMC7789568; doi:10.1186/s13148-020-00995-2)
Supplement: Supplementary file 4 — Additional file 4: Table S1. DMR lists at p < 1e−05 with presentation of name, chromosomal location, DMR start and stop nucleotide number for chromosome, length (bp), number of 1 kb significant windows, minimum p value, CpG number and density, DMR maximum, log-fold change (maxLFC) (+ increase DNA methylation and − decrease DNA methylation), and gene association within 10 kb and gene functional categories. [file 13148_2020_995_MOESM4_ESM.pdf]

Supplemental Table S1
DMR List Case vs. Control p<1e-05

DMR Name	Chr	Start	Stop	Length	# Sig Win	minP	maxLFC	CpG #	CpG Density	Gene Annotation	Gene Category
DMR1:386001	1	386001	389000	3000	1	6.10E-06	0.8139908	31	1.033	AL732372.2	
DMR1:517001	1	517001	518000	1000	1	3.58E-06	0.9372704	10	1	AL732372.2;RF00026	
DMR1:824001	1	824001	825000	1000	1	2.83E-07	-1.2835435	6	0.6	AL669831.3;AL669831.4;FAM87B;LINC01128;LINC00115	
DMR1:2656001	1	2656001	2667000	11000	3	1.10E-08	1.0405831	267	2.427	TTC34	
DMR1:2668001	1	2668001	2683000	15000	3	4.42E-08	0.8755558	363	2.42	TTC34	
DMR1:3571001	1	3571001	3572000	1000	1	1.60E-06	0.9307299	10	1	MEGF6	Growth Factors & Cytokines
DMR1:3920001	1	3920001	3921000	1000	1	2.77E-06	0.6803401	8	0.8	LINC01134	
DMR1:6258001	1	6258001	6259000	1000	1	5.33E-09	0.6549455	24	2.4	GPR153;ACOT7	Metabolism
DMR1:6522001	1	6522001	6523000	1000	1	9.22E-06	0.3394789	35	3.5	PLEKHG5;NOL9	Signaling;Transcription
DMR1:7222001	1	7222001	7224000	2000	1	7.86E-07	-0.4398214	34	1.7	CAMTA1;RNU1-8P	Transcription
DMR1:7966001	1	7966001	7967000	1000	1	4.45E-07	-0.571204	18	1.8	PARK7	Development
DMR1:9642001	1	9642001	9644000	2000	1	1.02E-06	0.705812	33	1.65	PIK3CD;PIK3CD-AS1	Signaling
DMR1:15866001	1	15866001	15867000	1000	1	4.64E-07	0.6211895	14	1.4	SPEN	Transcription
DMR1:19808001	1	19808001	19811000	3000	1	8.97E-06	-0.4319503	33	1.1	TMCO4;RNF186;AL391883.1	
DMR1:24159001	1	24159001	24162000	3000	1	3.90E-06	0.7355099	43	1.433	IFNLR1	Receptor
DMR1:24226001	1	24226001	24228000	2000	1	1.52E-06	0.6173313	26	1.3		
DMR1:26621001	1	26621001	26622000	1000	1	5.27E-06	0.7579737	69	6.9		
DMR1:28784001	1	28784001	28785000	1000	1	8.21E-06	0.5491186	25	2.5		
DMR1:28907001	1	28907001	28909000	2000	1	9.03E-07	0.5713446	44	2.2	EPB41	
DMR1:29480001	1	29480001	29481000	1000	1	6.96E-07	0.670635	10	1	AL671862.1	
DMR1:30010001	1	30010001	30011000	1000	1	7.09E-07	-0.5386825	20	2	LINC01648	
DMR1:33804001	1	33804001	33806000	2000	1	3.15E-07	-0.5887044	12	0.6	CSMD2	Unknown
DMR1:41502001	1	41502001	41503000	1000	1	9.96E-06	-0.5315307	9	0.9	HIVEP3	Transcription
DMR1:46799001	1	46799001	46800000	1000	1	8.31E-06	-0.5854623	13	1.3	CYP4B1	Metabolism
DMR1:54305001	1	54305001	54307000	2000	1	6.42E-06	-0.4431958	30	1.5	SSBP3	Translation
DMR1:59530001	1	59530001	59532000	2000	1	2.16E-06	-0.7317057	9	0.45	FGGY	Signaling
DMR1:61708001	1	61708001	61709000	1000	1	2.21E-06	0.8628242	6	0.6	TM2D1	Unknown
DMR1:69382001	1	69382001	69383000	1000	1	7.36E-06	-0.5941512	10	1		
DMR1:70268001	1	70268001	70269000	1000	1	5.34E-06	0.5314653	12	1.2	ANKRD13C	
DMR1:72494001	1	72494001	72495000	1000	1	8.03E-06	-0.625482	1	0.1		
DMR1:74449001	1	74449001	74450000	1000	1	1.94E-06	-0.4828113	8	0.8	FPGT-TNNI3K;TNNI3K	Transcription
DMR1:79458001	1	79458001	79459000	1000	1	2.48E-06	-0.5341593	9	0.9		
DMR1:79677001	1	79677001	79678000	1000	1	4.17E-06	-0.6074266	4	0.4		
DMR1:89368001	1	89368001	89369000	1000	1	6.02E-06	-0.6720726	6	0.6	GBP6	Signaling
DMR1:97504001	1	97504001	97506000	2000	1	4.69E-06	-0.8910787	12	0.6	DPYD	Metabolism
DMR1:103356001	1	103356001	103358000	2000	1	7.28E-08	-0.6163035	13	0.65		
DMR1:109039001	1	109039001	109040000	1000	1	5.02E-08	0.6790758	18	1.8	WDR47;BX679664.1;RANP5	
DMR1:109276001	1	109276001	109277000	1000	1	7.42E-07	0.8406167	15	1.5	CELSR2;PSRC1	Cytoskeleton
DMR1:109537001	1	109537001	109538000	1000	1	2.85E-06	0.6361615	13	1.3	GPR61;AL355310.3	Signaling
DMR1:112075001	1	112075001	112077000	2000	1	8.92E-07	0.7714121	5	0.25		
DMR1:115225001	1	115225001	115227000	2000	1	4.15E-06	-0.5591425	21	1.05		
DMR1:115384001	1	115384001	115385000	1000	1	2.98E-06	-0.9033439	1	0.1		
DMR1:116921001	1	116921001	116923000	2000	1	6.04E-06	-0.4536816	20	1	PTGFRN	
DMR1:117487001	1	117487001	117488000	1000	1	7.49E-08	-0.670835	6	0.6	MAN1A2;AL157902.2	Metabolism
DMR1:154605001	1	154605001	154607000	2000	1	9.58E-06	1.088414	23	1.15	ADAR	Transcription
DMR1:156166001	1	156166001	156167000	1000	1	3.15E-06	0.8921821	8	0.8	SEMA4A	Development
DMR1:156171001	1	156171001	156174000	3000	1	5.84E-06	0.5794197	48	1.6	SEMA4A	Development
DMR1:158269001	1	158269001	158270000	1000	1	2.47E-06	-0.5083161	10	1	HMGN1P5	
DMR1:161306001	1	161306001	161307000	1000	1	6.21E-07	1.1274576	35	3.5	MPZ;SDHC	Extracellular Matrix
DMR1:164199001	1	164199001	164200000	1000	1	4.12E-06	-0.4587256	7	0.7		
DMR1:166045001	1	166045001	166047000	2000	1	5.38E-06	-0.586517	11	0.55	RNA5SP64	
DMR1:170280001	1	170280001	170281000	1000	1	2.39E-06	-0.4550262	6	0.6	LINC01142	
DMR1:173100001	1	173100001	173101000	1000	1	1.46E-06	-0.5881991	5	0.5		
DMR1:173156001	1	173156001	173157000	1000	1	1.79E-07	-0.4918186	6	0.6		
DMR1:174089001	1	174089001	174090000	1000	1	3.08E-07	-0.5918141	5	0.5	RPL30P1	
DMR1:176750001	1	176750001	176751000	1000	1	7.81E-07	0.8580497	10	1	PAPPA2	
DMR1:178399001	1	178399001	178400000	1000	1	6.92E-06	-0.4565437	8	0.8	RASAL2	Signaling
DMR1:193063001	1	193063001	193064000	1000	1	9.19E-06	-0.6469959	6	0.6	UCHL5;SCARNA18B;RO60	Protease
DMR1:199919001	1	199919001	199920000	1000	1	7.47E-06	-0.4324768	10	1	AL445687.2	
DMR1:201006001	1	201006001	201008000	2000	1	1.27E-06	-0.3748639	22	1.1	KIF21B	Cytoskeleton
DMR1:201696001	1	201696001	201697000	1000	1	5.80E-06	-0.5161451	23	2.3	NAV1;IPO9-AS1	
DMR1:204002001	1	204002001	204003000	1000	1	4.95E-06	0.9292689	10	1		
DMR1:205229001	1	205229001	205231000	2000	1	2.97E-07	-0.7229921	46	2.3	TMCC2;AC093422.2	Unknown
DMR1:206732001	1	206732001	206734000	2000	1	7.16E-06	-0.3181144	39	1.95	MAPKAPK2	Signaling
DMR1:207843001	1	207843001	207844000	1000	1	6.98E-06	-0.4149983	12	1.2	MIR29B2CHG	

DMR1:211276001	1	211276001	211278000	2000	1	3.56E-06	0.8699388	21	1.05	RCOR3	Transcription
DMR1:212661001	1	212661001	212662000	1000	1	1.20E-06	-0.6404967	4	0.4	AL590648.3	
DMR1:224610001	1	224610001	224611000	1000	1	5.04E-06	-0.6638086	6	0.6	CNIH3;AC096537.1	Signaling
DMR1:231394001	1	231394001	231396000	2000	1	7.09E-06	-0.4773044	27	1.35	EGLN1	Translation
DMR1:236668001	1	236668001	236670000	2000	1	1.89E-06	0.6024508	40	2	ACTN2	Cytoskeleton
DMR1:236985001	1	236985001	236987000	2000	1	3.72E-06	-0.466462	19	0.95	RPL35P1	
DMR1:246002001	1	246002001	246003000	1000	1	8.84E-06	0.5305773	56	5.6	SMYD3	Epigenetic
DMR2:971001	2	971001	973000	2000	1	3.43E-06	-0.6292529	4	0.2	SNTG2	Cytoskeleton
DMR2:5884001	2	5884001	5885000	1000	1	6.04E-07	0.9563322	36	3.6		
DMR2:7062001	2	7062001	7063000	1000	1	3.34E-06	-0.8149463	6	0.6	RNF144A;AC068481.1	Metabolism
DMR2:7085001	2	7085001	7086000	1000	1	8.12E-06	-0.9066546	3	0.3	AC068481.1	
DMR2:12582001	2	12582001	12583000	1000	1	1.19E-06	-0.5846639	8	0.8	MIR3681HG	
DMR2:14247001	2	14247001	14248000	1000	1	7.65E-07	-0.6482546	7	0.7	LINC00276	
DMR2:15680001	2	15680001	15682000	2000	1	1.07E-06	0.9947209	72	3.6	AC008278.1;LINC01804	
DMR2:17995001	2	17995001	17996000	1000	1	6.32E-08	-0.8903978	6	0.6	KCNS3	
DMR2:23421001	2	23421001	23423000	2000	1	5.83E-06	-0.2908985	15	0.75	KLHL29	Transcription
DMR2:23688001	2	23688001	23689000	1000	1	3.21E-06	-0.5998376	18	1.8	KLHL29;AC009242.1	Transcription
DMR2:29192001	2	29192001	29193000	1000	1	4.22E-06	-0.9522174	11	1.1	CLIP4;ALK	Cytoskeleton;Receptor
DMR2:29413001	2	29413001	29416000	3000	1	5.16E-06	0.7047872	31	1.033	ALK	Receptor
DMR2:30565001	2	30565001	30567000	2000	1	6.12E-06	-0.5461257	20	1	LCLAT1	Metabolism
DMR2:37892001	2	37892001	37893000	1000	1	2.24E-07	-0.561919	11	1.1		
DMR2:42592001	2	42592001	42593000	1000	1	8.07E-06	0.6667141	12	1.2	MTA3	Transcription
DMR2:43833001	2	43833001	43835000	2000	1	1.00E-06	0.4922924	31	1.55	ABCG5;ABCG8	Transport
DMR2:47317001	2	47317001	47319000	2000	1	7.72E-06	-0.4133311	36	1.8	EPCAM-DT	
DMR2:62190001	2	62190001	62191000	1000	1	5.77E-06	-0.4406462	4	0.4	B3GNT2	Golgi
DMR2:65396001	2	65396001	65398000	2000	1	1.00E-06	-0.6272254	13	0.65	SPRED2	Signaling
DMR2:67152001	2	67152001	67154000	2000	1	6.64E-06	-0.8184915	9	0.45	LINC01828;LINC01829	
DMR2:71320001	2	71320001	71322000	2000	1	2.80E-07	-0.6199767	11	0.55	ZNF638	Transcription
DMR2:85381001	2	85381001	85382000	1000	1	6.53E-06	0.6174528	12	1.2	ELMOD3;AC062037.2	Signaling
DMR2:85861001	2	85861001	85862000	1000	1	2.62E-06	-0.9205441	7	0.7	ST3GAL5	Metabolism
DMR2:95774001	2	95774001	95776000	2000	1	9.60E-06	-0.9386901	28	1.4		
DMR2:97114001	2	97114001	97115000	1000	1	8.24E-09	1.528267	20	2	ANKRD36	
DMR2:97700001	2	97700001	97701000	1000	1	9.64E-06	0.3839572	27	2.7	C2orf92	
DMR2:103661001	2	103661001	103662000	1000	1	8.41E-06	-0.7608563	4	0.4		
DMR2:108939001	2	108939001	108940000	1000	1	4.85E-06	0.6210809	13	1.3	EDAR	
DMR2:109605001	2	109605001	109607000	2000	1	1.55E-06	0.6797456	21	1.05	SEPT10;AC011753.2;SOWAHC	Cytoskeleton
DMR2:112013001	2	112013001	112014000	1000	1	9.34E-06	-0.487958	10	1	MERTK	Signaling
DMR2:117479001	2	117479001	117480000	1000	1	3.73E-06	0.8066244	8	0.8		
DMR2:121190001	2	121190001	121191000	1000	1	5.63E-06	0.4716109	21	2.1	AC079988.1	
DMR2:121724001	2	121724001	121725000	1000	1	6.29E-06	0.4424856	21	2.1	NIFK-AS1;NIFK	
DMR2:121968001	2	121968001	121970000	2000	1	5.54E-07	-0.5120706	19	0.95		
DMR2:126173001	2	126173001	126175000	2000	1	2.47E-06	-0.3616873	24	1.2		
DMR2:126934001	2	126934001	126936000	2000	1	1.33E-07	-0.5608458	25	1.25		
DMR2:127269001	2	127269001	127271000	2000	1	4.39E-06	0.5781201	29	1.45	ERCC3	Transcription
DMR2:127384001	2	127384001	127385000	1000	1	2.97E-06	0.5969059	18	1.8	AC068282.1;MAP3K2;MAP3K2-DT	Signaling
DMR2:133138001	2	133138001	133139000	1000	1	4.48E-08	-0.5614005	9	0.9	NCKAP5	
DMR2:133188001	2	133188001	133191000	3000	1	4.21E-06	-0.5582199	16	0.533	NCKAP5	
DMR2:134588001	2	134588001	134590000	2000	1	9.46E-07	-0.5403289	11	0.55	TMEM163	Unknown
DMR2:149512001	2	149512001	149513000	1000	1	9.39E-06	-0.5100184	12	1.2		
DMR2:150326001	2	150326001	150327000	1000	1	7.55E-07	0.9147427	8	0.8		
DMR2:158353001	2	158353001	158355000	2000	1	6.66E-06	-0.4803046	15	0.75	CCDC148	
DMR2:158494001	2	158494001	158496000	2000	1	6.42E-06	0.583786	25	1.25	PKP4	Cytoskeleton
DMR2:159064001	2	159064001	159065000	1000	1	7.45E-06	-0.7027179	7	0.7	TANC1;GSTM3P2	Unknown
DMR2:165874001	2	165874001	165875000	1000	1	1.67E-07	1.0363303	13	1.3	TTC21B	Unknown
DMR2:167435001	2	167435001	167437000	2000	1	8.76E-06	-0.4750665	16	0.8	AC073050.1	
DMR2:171896001	2	171896001	171898000	2000	1	8.34E-06	0.5058302	24	1.2	SLC25A12	Binding Protein
DMR2:175484001	2	175484001	175486000	2000	1	4.77E-06	-0.5590207	16	0.8		
DMR2:175985001	2	175985001	175986000	1000	1	7.98E-06	0.480612	9	0.9	LNPK	
DMR2:178942001	2	178942001	178943000	1000	1	4.05E-06	-0.707268	6	0.6	CCDC141	Cytoskeleton
DMR2:179049001	2	179049001	179050000	1000	1	9.66E-06	-0.5780055	11	1.1	CCDC141	Cytoskeleton
DMR2:179274001	2	179274001	179276000	2000	1	6.99E-06	-0.5545355	8	0.4	SESTD1;AC093911.1	Signaling
DMR2:181285001	2	181285001	181286000	1000	1	1.79E-06	-0.8246201	6	0.6	LINC01934	
DMR2:185552001	2	185552001	185553000	1000	1	1.49E-10	1.3364364	6	0.6	AC080125.1	
DMR2:186988001	2	186988001	186989000	1000	1	8.25E-06	-0.4016445	15	1.5		
DMR2:188879001	2	188879001	188880000	1000	1	5.56E-06	-0.5148295	6	0.6		
DMR2:189185001	2	189185001	189187000	2000	1	8.58E-06	1.0300489	23	1.15	COL5A2	Extracellular Matrix
DMR2:189715001	2	189715001	189716000	1000	1	8.93E-06	-0.6476778	7	0.7	ANKAR	
DMR2:194639001	2	194639001	194640000	1000	1	7.12E-06	0.6004123	18	1.8		
DMR2:202974001	2	202974001	202975000	1000	1	3.97E-06	0.5237985	17	1.7	WDR12;CARF	Unknown
DMR2:203259001	2	203259001	203260000	1000	1	9.72E-06	0.5921851	14	1.4	CYP20A1;RN7SL670P	Electron Transport

DMR2:204061001	2	204061001	204062000	1000	1	1.33E-09	-0.7914435	9	0.9	AC009965.2	
DMR2:206667001	2	206667001	206669000	2000	1	3.27E-08	-0.7418124	15	0.75	DYTN	
DMR2:208215001	2	208215001	208216000	1000	1	8.26E-06	0.6347569	15	1.5	RPSAP27;TPT1P2	
DMR2:211716001	2	211716001	211717000	1000	1	2.11E-06	0.4703591	30	3	ERBB4	Signaling
DMR2:216710001	2	216710001	216713000	3000	1	1.59E-07	-0.5847288	39	1.3	AC007563.2	
DMR2:218561001	2	218561001	218562000	1000	1	4.87E-07	-0.4202055	12	1.2	USP37;CNOT9	Protease
DMR2:226806001	2	226806001	226808000	2000	1	7.02E-07	-0.6563554	15	0.75	IRS1;AC010735.2;AC010735.1	Unknown
DMR2:231272001	2	231272001	231273000	1000	1	2.75E-06	0.5428854	20	2	ARMC9	
DMR2:235963001	2	235963001	235965000	2000	1	3.57E-07	-0.5756016	36	1.8	AGAP1	Signaling
DMR3:304001	3	304001	305000	1000	1	2.59E-06	-0.662561	7	0.7	CHL1;RPS8P6	Extracellular Matrix
DMR3:1034001	3	1034001	1035000	1000	1	1.10E-07	-0.9882	5	0.5		
DMR3:9344001	3	9344001	9345000	1000	1	7.73E-07	0.5947837	25	2.5	SRGAP3;AC026191.1;PGAM1P4;THUMPD3-AS1	Signaling
DMR3:12368001	3	12368001	12370000	2000	1	3.30E-06	0.7976002	17	0.85	PPARG	Receptor
DMR3:14334001	3	14334001	14335000	1000	1	2.11E-07	-0.5358308	12	1.2		
DMR3:15593001	3	15593001	15596000	3000	1	8.85E-06	0.6773648	45	1.5	HACL1;BTD	Metabolism
DMR3:19593001	3	19593001	19594000	1000	1	9.42E-06	-0.379851	9	0.9		
DMR3:20600001	3	20600001	20601000	1000	1	6.74E-07	-0.5380999	10	1	SGO1-AS1	
DMR3:32765001	3	32765001	32767000	2000	1	5.67E-06	1.1020184	36	1.8	CNOT10	
DMR3:34173001	3	34173001	34174000	1000	1	8.35E-06	-0.733452	4	0.4	LINC01811	
DMR3:46373001	3	46373001	46376000	3000	1	3.79E-07	0.7776259	30	1	AC098613.1;CCR5	Growth Factors & Cytokines
DMR3:48201001	3	48201001	48202000	1000	1	9.15E-06	0.5055743	29	2.9	MIR4443	
DMR3:50192001	3	50192001	50193000	1000	1	6.23E-06	1.3249619	25	2.5	SEMA3F;GNAT1	Growth Factors & Cytokines;Signaling
DMR3:51110001	3	51110001	51111000	1000	1	6.32E-06	-0.5088342	4	0.4	DOCK3	Signaling
DMR3:54078001	3	54078001	54079000	1000	1	9.12E-06	-0.9024898	10	1		
DMR3:59570001	3	59570001	59572000	2000	1	1.87E-06	0.5355753	16	0.8		
DMR3:64181001	3	64181001	64183000	2000	1	2.93E-06	-0.5960076	13	0.65	PRICKLE2;PRDX3P4;PRICKLE2-AS3	Cytoskeleton
DMR3:68392001	3	68392001	68394000	2000	1	4.11E-06	-0.5435105	15	0.75	FAM19A1	
DMR3:73021001	3	73021001	73022000	1000	1	5.31E-06	0.9559614	6	0.6	PPP4R2;RNU7-19P	
DMR3:74120001	3	74120001	74121000	1000	1	2.34E-08	-1.4543578	5	0.5		
DMR3:78490001	3	78490001	78491000	1000	1	7.61E-09	-0.6011024	11	1.1		
DMR3:87649001	3	87649001	87651000	2000	1	2.10E-06	-0.8331352	8	0.4	AC108749.1	
DMR3:88836001	3	88836001	88837000	1000	1	9.61E-07	-0.9823663	6	0.6		
DMR3:89157001	3	89157001	89158000	1000	1	2.80E-07	-1.0778311	8	0.8	EPHA3	Receptor
DMR3:91375001	3	91375001	91376000	1000	1	3.91E-07	1.1716095	3	0.3	ABBA01000935.2	
DMR3:91549001	3	91549001	91554000	5000	2	7.47E-07	1.0363492	79	1.58		
DMR3:93705001	3	93705001	93714000	9000	3	3.57E-08	0.8443159	153	1.7		
DMR3:100255001	3	100255001	100256000	1000	1	1.04E-07	1.0378051	5	0.5	TBC1D23	
DMR3:100289001	3	100289001	100290000	1000	1	7.50E-06	0.8356997	9	0.9	TBC1D23	
DMR3:110714001	3	110714001	110715000	1000	1	5.50E-06	-0.9557768	5	0.5		
DMR3:117669001	3	117669001	117670000	1000	1	1.41E-06	-0.7118383	10	1	AC092691.1;AC092691.2;LINC02024	
DMR3:122419001	3	122419001	122420000	1000	1	2.99E-07	0.6840997	40	4	FAM162A;WDR5B;AC083798.2;AC083798.1;KPNA1	Unknown;Metabolism
DMR3:129294001	3	129294001	129295000	1000	1	3.67E-07	0.6314323	16	1.6	HMCES	
DMR3:130403001	3	130403001	130405000	2000	1	3.20E-06	-0.8355836	9	0.45	COL6A5	
DMR3:134740001	3	134740001	134741000	1000	1	1.67E-06	0.5250261	21	2.1	EPHB1	Receptor
DMR3:139029001	3	139029001	139030000	1000	1	8.83E-07	-0.4752568	9	0.9	MRPS22;PRR23B	Transcription
DMR3:143232001	3	143232001	143233000	1000	1	9.77E-06	-0.7744006	3	0.3		
DMR3:151846001	3	151846001	151847000	1000	1	7.08E-06	-0.8926749	13	1.3	AADACL2-AS1	
DMR3:155545001	3	155545001	155546000	1000	1	4.90E-06	-0.4916974	7	0.7	PLCH1	Metabolism
DMR3:159021001	3	159021001	159022000	1000	1	3.82E-07	-1.0881578	2	0.2	IQCJ-SCHIP1;IQCJ	
DMR3:168445001	3	168445001	168449000	4000	1	1.36E-06	-0.8900465	22	0.55	EGFEM1P	
DMR3:170743001	3	170743001	170744000	1000	1	4.53E-06	-0.6188246	7	0.7	AC026316.4;SLC7A14-AS1;AC026316.2	
DMR3:181107001	3	181107001	181108000	1000	1	9.79E-06	-0.5737711	13	1.3	SOX2-OT	
DMR3:184810001	3	184810001	184811000	1000	1	5.88E-06	0.774526	8	0.8	VPS8	
DMR3:185345001	3	185345001	185346000	1000	1	5.97E-06	-0.3851038	11	1.1	MAP3K13	Signaling
DMR3:185844001	3	185844001	185845000	1000	1	3.88E-06	-0.6385158	11	1.1		
DMR3:188581001	3	188581001	188582000	1000	1	6.75E-07	-0.4045768	8	0.8	LPP	Cytoskeleton
DMR3:192432001	3	192432001	192434000	2000	1	2.43E-06	-0.5818597	20	1	FGF12	Growth Factors & Cytokines
DMR3:196945001	3	196945001	196946000	1000	1	9.74E-06	0.5237731	20	2	NCBP2;NCBP2-AS1;NCBP2-AS2;PIGZ	Transcription;Metabolism
DMR4:3759001	4	3759001	3760000	1000	1	6.31E-06	-0.5600381	29	2.9	LINC02600;ADRA2C	Receptor
DMR4:6951001	4	6951001	6953000	2000	1	1.99E-06	-0.4179546	26	1.3	TBC1D14	Signaling
DMR4:7448001	4	7448001	7451000	3000	1	9.05E-06	0.8706796	45	1.5	SORCS2;MIR4274	Receptor
DMR4:9862001	4	9862001	9863000	1000	1	2.44E-06	-0.708581	8	0.8	SLC2A9	Metabolism
DMR4:10066001	4	10066001	10067000	1000	1	5.57E-07	-0.5455646	11	1.1	AC005674.2;WDR1	Unknown
DMR4:12097001	4	12097001	12098000	1000	1	7.77E-06	-0.8038681	4	0.4		
DMR4:13176001	4	13176001	13177000	1000	1	1.61E-06	-0.5710104	8	0.8		
DMR4:16890001	4	16890001	16891000	1000	1	5.75E-07	-0.7572532	10	1	LDB2	Transcription
DMR4:18209001	4	18209001	18212000	3000	1	2.17E-07	-0.6757687	11	0.367		

DMR4:49222001	4	49222001	49223000	1000	1	1.02E-06	0.5127587	6	0.6	AC118282.2;AC118282.4	
DMR4:49224001	4	49224001	49226000	2000	1	4.26E-07	0.611876	15	0.75	AC118282.4;SNX18P23	
DMR4:52666001	4	52666001	52667000	1000	1	3.84E-06	0.5704389	2	0.2	USP46;USP46-AS1	Proteolysis
DMR4:52973001	4	52973001	52974000	1000	1	5.63E-06	-0.6395201	5	0.5	SCFD2	Unknown
DMR4:53467001	4	53467001	53469000	2000	1	5.10E-06	-0.7313163	22	1.1	FIP1L1;AC058822.1;LNX1	Cytoskeleton
DMR4:63099001	4	63099001	63100000	1000	1	1.24E-06	-0.6469376	4	0.4		
DMR4:72705001	4	72705001	72706000	1000	1	1.95E-06	0.7554899	12	1.2		
DMR4:75551001	4	75551001	75552000	1000	1	6.14E-07	0.5856654	18	1.8	THAP6;ODAPH	Transcription
DMR4:76025001	4	76025001	76026000	1000	1	3.02E-06	-0.5129691	7	0.7	ART3;CXCL10;CXCL11	Metabolism;Growth Factors & Cytokines
DMR4:87346001	4	87346001	87347000	1000	1	6.15E-07	0.7495587	15	1.5	AC108516.2;HSD17B11	Metabolism
DMR4:88979001	4	88979001	88980000	1000	1	5.00E-06	0.7908192	8	0.8	FAM13A	
DMR4:94591001	4	94591001	94593000	2000	1	1.71E-06	-0.7403106	15	0.75	PDLIM5	Cytoskeleton
DMR4:98055001	4	98055001	98057000	2000	1	2.10E-06	-0.8233235	13	0.65	STPG2;DUTP8	Development
DMR4:98282001	4	98282001	98284000	2000	1	2.73E-07	-0.5139398	14	0.7	RAP1GDS1	Signaling
DMR4:99064001	4	99064001	99066000	2000	1	1.24E-06	0.8542533	22	1.1	METAP1;AC019131.2;ADH5	Protease;Metabolism
DMR4:100090001	4	100090001	100091000	1000	1	1.58E-06	-0.6226641	4	0.4	AC097460.1	
DMR4:100204001	4	100204001	100205000	1000	1	1.90E-06	0.9039055	21	2.1	AC097460.1;AP001961.1	
DMR4:104066001	4	104066001	104067000	1000	1	2.28E-07	-0.8211243	9	0.9		
DMR4:107484001	4	107484001	107485000	1000	1	3.40E-07	-0.6626713	5	0.5		
DMR4:122751001	4	122751001	122752000	1000	1	1.91E-06	-0.5261898	6	0.6	BBS12	
DMR4:128916001	4	128916001	128917000	1000	1	7.29E-06	-0.905045	6	0.6	SCLT1	Metabolism
DMR4:140013001	4	140013001	140015000	2000	1	6.49E-06	-0.8846538	14	0.7	MAML3	EST
DMR4:143686001	4	143686001	143687000	1000	1	2.54E-06	-0.5027995	10	1	AC107223.1;FREM3	Transport
DMR4:147213001	4	147213001	147215000	2000	1	2.20E-06	-0.8128533	8	0.4		
DMR4:153111001	4	153111001	153112000	1000	1	2.55E-06	-0.6121456	14	1.4		
DMR4:164321001	4	164321001	164322000	1000	1	2.74E-06	-0.7249916	5	0.5	1-Mar	Metabolism
DMR4:165109001	4	165109001	165111000	2000	1	9.92E-06	0.7641435	19	0.95	TMEM192	Unknown
DMR4:184480001	4	184480001	184481000	1000	1	1.01E-06	-0.4423459	6	0.6	IRF2;AC099343.2;AC099343.3	Transcription
DMR4:188636001	4	188636001	188637000	1000	1	4.63E-06	0.681319	15	1.5	LINC01060;AC093909.3	
DMR5:696001	5	696001	699000	3000	1	2.05E-06	-0.6667814	48	1.6	TPPP	Cytoskeleton
DMR5:1461001	5	1461001	1463000	2000	1	6.49E-06	-1.7427536	53	2.65	LPCAT1	Metabolism
DMR5:3128001	5	3128001	3130000	2000	1	3.94E-06	-0.6158025	51	2.55		
DMR5:4492001	5	4492001	4493000	1000	1	7.33E-08	-0.7392452	15	1.5	AC106799.2	
DMR5:20445001	5	20445001	20447000	2000	1	4.42E-06	-0.5958123	13	0.65	CDH18	Cytoskeleton
DMR5:27224001	5	27224001	27225000	1000	1	1.94E-08	-0.8761978	5	0.5	PURPL	
DMR5:32070001	5	32070001	32071000	1000	1	1.02E-06	0.6963497	17	1.7	PDZD2	
DMR5:37976001	5	37976001	37978000	2000	1	5.29E-06	-0.7481109	16	0.8	AC034226.1	
DMR5:39122001	5	39122001	39123000	1000	1	8.38E-06	-0.4437573	7	0.7	FYB1	
DMR5:44758001	5	44758001	44759000	1000	1	9.05E-06	-0.4511642	9	0.9	MRPS30-DT;AC093297.1	
DMR5:46433001	5	46433001	46436000	3000	1	4.69E-06	-0.5484316	37	1.233		
DMR5:54849001	5	54849001	54850000	1000	1	9.09E-08	-0.7133944	8	0.8	AC112198.2;AC112198.1	
DMR5:56154001	5	56154001	56155000	1000	1	2.30E-06	0.5844628	17	1.7	ANKRD55;RNA5SP184	
DMR5:57379001	5	57379001	57380000	1000	1	1.46E-07	-0.5826456	10	1		
DMR5:62675001	5	62675001	62676000	1000	1	4.01E-10	-0.5902725	6	0.6		
DMR5:65984001	5	65984001	65985000	1000	1	3.74E-06	-0.5831688	12	1.2	ERBIN	
DMR5:75179001	5	75179001	75180000	1000	1	2.07E-06	-0.5246361	6	0.6	ANKRD31	
DMR5:79263001	5	79263001	79264000	1000	1	1.58E-06	0.5879923	18	1.8	JMY	
DMR5:80306001	5	80306001	80308000	2000	1	3.68E-07	0.6320542	34	1.7	AC026410.1;RF00322;AC026410.2	
DMR5:87198001	5	87198001	87199000	1000	1	9.41E-06	0.4671994	12	1.2	LINC01949	
DMR5:94045001	5	94045001	94046000	1000	1	4.21E-06	0.8850949	3	0.3	FAM172A	Unknown
DMR5:102044001	5	102044001	102045000	1000	1	1.42E-06	-0.4686804	8	0.8		
DMR5:105574001	5	105574001	105575000	1000	1	9.89E-06	0.7868771	12	1.2		
DMR5:105598001	5	105598001	105599000	1000	1	7.36E-06	-0.6168524	7	0.7		
DMR5:122279001	5	122279001	122280000	1000	1	9.77E-06	-0.8489853	3	0.3		
DMR5:126188001	5	126188001	126189000	1000	1	1.50E-08	-1.0559611	8	0.8	AC116362.1;LINC02039	
DMR5:126715001	5	126715001	126716000	1000	1	3.10E-06	0.5800275	7	0.7		
DMR5:133134001	5	133134001	133136000	2000	1	8.76E-06	-0.55855	10	0.5		
DMR5:134757001	5	134757001	134758000	1000	1	6.46E-09	1.082406	12	1.2	CAMLG;DDX46	Transcription
DMR5:135090001	5	135090001	135091000	1000	1	7.16E-06	-0.6150929	3	0.3	C5orf66	
DMR5:135780001	5	135780001	135781000	1000	1	5.96E-06	1.1879249	25	2.5	SLC25A48	
DMR5:138562001	5	138562001	138564000	2000	1	1.95E-06	0.5819006	26	1.3	HSPA9;SNORD63B;SNORD63	Signaling
DMR5:139469001	5	139469001	139470000	1000	1	2.22E-06	-0.5321188	10	1	AC142391.1;ECSCR;SMIM33;TMEM173	
DMR5:139858001	5	139858001	139860000	2000	1	1.17E-06	0.4361855	21	1.05	NRG2;AC008667.2	Signaling
DMR5:141114001	5	141114001	141115000	1000	1	4.39E-07	-0.4926117	6	0.6	AC244517.2;AC244517.1;PCDHB4	Cytoskeleton
DMR5:160271001	5	160271001	160272000	1000	1	1.26E-06	-0.533452	19	1.9	CCNJL	Signaling
DMR5:164170001	5	164170001	164171000	1000	1	4.13E-06	-0.6355043	8	0.8	AC008662.1	
DMR5:165244001	5	165244001	165245000	1000	1	2.19E-06	-0.5904441	6	0.6	LINC01938	
DMR5:173415001	5	173415001	173417000	2000	1	3.69E-06	-0.427507	15	0.75	AC016573.1	
DMR5:173729001	5	173729001	173730000	1000	1	3.40E-06	-0.8118145	12	1.2	LINC01484	
DMR5:175062001	5	175062001	175063000	1000	1	6.94E-07	-0.4382482	11	1.1		

DMR5:177954001	5	177954001	177955000	1000	1	8.84E-06	0.5201743	14	1.4	AC106795.1;AC106795.3;AC106795.2	
DMR5:179132001	5	179132001	179138000	6000	1	8.10E-06	0.9730536	168	2.8	ADAMTS2	Protease
DMR5:179969001	5	179969001	179970000	1000	1	5.15E-06	0.6155863	15	1.5	RNF130;AC010285.1;AC010285.3	Metabolism
DMR5:180779001	5	180779001	180780000	1000	1	3.90E-06	-0.4453688	12	1.2	MGAT1	Metabolism
DMR6:1402001	6	1402001	1404000	2000	2	2.66E-06	-0.4116056	34	1.7	FOXF2	Transcription
DMR6:3924001	6	3924001	3925000	1000	1	3.73E-06	-0.5157897	13	1.3	AL590004.2;AL590004.1	
DMR6:6430001	6	6430001	6431000	1000	1	9.25E-06	-0.5263292	5	0.5	LY86-AS1	
DMR6:6718001	6	6718001	6721000	3000	1	1.16E-06	-0.8640551	34	1.133	AL031123.1	
DMR6:11738001	6	11738001	11741000	3000	1	6.40E-06	-0.7211799	27	0.9	ADTRP	Development
DMR6:12921001	6	12921001	12922000	1000	1	1.52E-06	0.9800264	2	0.2	PHACTR1	Signaling
DMR6:13133001	6	13133001	13134000	1000	1	7.27E-06	0.602249	51	5.1	PHACTR1	Signaling
DMR6:14622001	6	14622001	14623000	1000	1	5.64E-06	-0.4587547	22	2.2		
DMR6:19698001	6	19698001	19699000	1000	1	6.34E-07	-0.3771312	17	1.7	AL022068.1	
DMR6:21055001	6	21055001	21056000	1000	1	2.87E-06	-1.1051213	3	0.3	CDKAL1	Cell Cycle
DMR6:24039001	6	24039001	24041000	2000	1	8.51E-06	-0.9233759	7	0.35		
DMR6:28926001	6	28926001	28927000	1000	1	8.45E-06	0.8705091	15	1.5	TRIM27	Metabolism
DMR6:29618001	6	29618001	29619000	1000	1	1.19E-06	-0.636553	6	0.6	GABBR1	Receptor
DMR6:38496001	6	38496001	38497000	1000	1	1.42E-06	-0.7941924	7	0.7	BTBD9	Unknown
DMR6:38624001	6	38624001	38625000	1000	1	2.89E-07	-0.6006061	15	1.5	BTBD9	Unknown
DMR6:43546001	6	43546001	43547000	1000	1	6.01E-06	-1.0896769	5	0.5	POLR1C;XPO5;AL355802.1;RF00426	Translation;Receptor
DMR6:43856001	6	43856001	43857000	1000	1	4.81E-07	-1.1220187	6	0.6	LINC02537;AL157371.1	
DMR6:49550001	6	49550001	49551000	1000	1	9.52E-07	0.8714945	49	4.9	C6orf141	
DMR6:52624001	6	52624001	52625000	1000	1	6.99E-06	0.7442294	16	1.6		
DMR6:79220001	6	79220001	79221000	1000	1	4.37E-06	0.6615165	9	0.9	HMGN3	Epigenetic
DMR6:89472001	6	89472001	89473000	1000	1	7.35E-06	-0.7215289	11	1.1	ANKRD6;RN7SL11P	
DMR6:91335001	6	91335001	91336000	1000	1	4.31E-06	-0.5591004	7	0.7		
DMR6:97192001	6	97192001	97193000	1000	1	4.66E-06	1.016525	3	0.3	MMS22L	
DMR6:99166001	6	99166001	99168000	2000	1	9.50E-07	-0.6259245	9	0.45	BDH2P1	
DMR6:99861001	6	99861001	99862000	1000	1	8.83E-09	-1.0353924	3	0.3		
DMR6:102673001	6	102673001	102675000	2000	1	9.49E-08	-0.6446693	12	0.6		
DMR6:104679001	6	104679001	104680000	1000	1	2.88E-06	0.7593947	6	0.6	AL356967.1	
DMR6:110799001	6	110799001	110801000	2000	1	1.52E-06	-0.6955559	14	0.7	CDK19	Signaling
DMR6:119104001	6	119104001	119105000	1000	1	1.35E-06	1.016355	8	0.8	AL137009.1;FAM184A	Unknown
DMR6:119412001	6	119412001	119414000	2000	1	6.30E-06	-0.7922276	6	0.3		
DMR6:119589001	6	119589001	119590000	1000	1	3.17E-06	-0.4975542	3	0.3		
DMR6:122964001	6	122964001	122965000	1000	1	8.16E-06	-1.0593466	1	0.1		
DMR6:124759001	6	124759001	124761000	2000	1	5.75E-07	0.6666712	21	1.05	NKAIN2	Transport
DMR6:135329001	6	135329001	135332000	3000	1	3.71E-06	-0.869098	25	0.833	AHI1	Development
DMR6:150690001	6	150690001	150691000	1000	1	9.60E-06	-0.5202252	10	1	PLEKHG1	Signaling
DMR6:151058001	6	151058001	151059000	1000	1	2.29E-06	0.6011066	16	1.6	MTHFD1L;AL133260.2	Metabolism
DMR6:154006001	6	154006001	154007000	1000	1	3.02E-06	-0.7391696	3	0.3	OPRM1	Receptor
DMR6:156358001	6	156358001	156360000	2000	1	3.95E-06	-0.435247	27	1.35	AL512658.2	
DMR6:159658001	6	159658001	159659000	1000	1	4.31E-07	-0.510362	5	0.5		
DMR6:159707001	6	159707001	159709000	2000	1	2.36E-07	-0.6397778	13	0.65	SOD2;HNRNPH1P1	Metabolism
DMR6:160945001	6	160945001	160948000	3000	1	2.05E-06	-0.4429917	33	1.1	AL139393.1	
DMR6:167422001	6	167422001	167424000	2000	1	5.02E-07	0.7600747	143	7.15		
DMR7:1273001	7	1273001	1279000	6000	1	5.10E-06	0.5570609	200	3.333	AC073094.1	
DMR7:2902001	7	2902001	2904000	2000	1	1.27E-07	0.7713864	35	1.75	CARD11	Unknown
DMR7:7648001	7	7648001	7650000	2000	1	5.29E-07	-0.6083148	20	1	RPA3;UMAD1;AC007161.3	Cytoskeleton
DMR7:17916001	7	17916001	17918000	2000	1	1.28E-06	-0.6276699	13	0.65	SNX13	Signaling
DMR7:26081001	7	26081001	26083000	2000	1	1.79E-06	-0.7509067	14	0.7		
DMR7:28416001	7	28416001	28418000	2000	1	4.34E-06	-0.53156	16	0.8	CREB5	Transcription
DMR7:30512001	7	30512001	30514000	2000	1	8.02E-06	-0.4580246	20	1	GGCT;AC005154.5;GARS-DT;AC005154.2	
DMR7:32601001	7	32601001	32602000	1000	1	1.07E-06	-0.6311666	8	0.8	DPY19L1P1	
DMR7:35347001	7	35347001	35348000	1000	1	6.54E-06	0.8059713	12	1.2		
DMR7:35750001	7	35750001	35751000	1000	1	3.61E-06	-0.456453	5	0.5	SEPT7-AS1	
DMR7:37123001	7	37123001	37124000	1000	1	2.27E-07	-0.5588348	5	0.5	ELMO1;RPS17P13	Signaling
DMR7:37689001	7	37689001	37691000	2000	1	9.39E-06	-0.4653141	19	0.95	GPR141;EPDR1	Receptor
DMR7:38672001	7	38672001	38673000	1000	1	8.17E-06	-0.5160182	12	1.2		
DMR7:40481001	7	40481001	40482000	1000	1	4.84E-07	1.0367547	9	0.9	SUGCT	EST
DMR7:42733001	7	42733001	42734000	1000	1	5.91E-09	-0.6650931	10	1		
DMR7:42927001	7	42927001	42929000	2000	1	6.42E-06	0.7891598	19	0.95	AC010132.3;PSMA2;AC010132.2;MRPL32	
DMR7:44024001	7	44024001	44026000	2000	1	3.41E-06	0.7581505	18	0.9	POLR2J4;AC004951.4;AC017116.2;RASA4CP	
DMR7:44804001	7	44804001	44805000	1000	1	7.50E-06	0.6919024	13	1.3	PPIA	
DMR7:46116001	7	46116001	46117000	1000	1	1.39E-06	-0.5695176	8	0.8		
DMR7:50060001	7	50060001	50061000	1000	1	2.21E-06	-0.6013637	5	0.5	ZPBP	Development
DMR7:50814001	7	50814001	50815000	1000	1	5.01E-06	0.5820185	11	1.1		
DMR7:53275001	7	53275001	53276000	1000	1	2.40E-06	0.6137477	9	0.9		

DMR7:65521001	7	65521001	65522000	1000	1	6.72E-06	-0.8485457	9	0.9	AC114501.2	
DMR7:68057001	7	68057001	68058000	1000	1	8.28E-06	0.7511854	9	0.9		
DMR7:72788001	7	72788001	72791000	3000	1	9.28E-06	0.5741053	42	1.4	TYW1B	
DMR7:73698001	7	73698001	73699000	1000	1	3.21E-06	0.5988557	12	1.2	BUD23;STX1A	Metabolism;Transport
DMR7:74011001	7	74011001	74012000	1000	1	1.47E-06	0.7238667	18	1.8		
DMR7:74096001	7	74096001	74097000	1000	1	5.46E-06	0.5571859	22	2.2	LIMK1	Signaling
DMR7:98826001	7	98826001	98827000	1000	1	7.36E-06	-0.4244376	31	3.1		
DMR7:103693001	7	103693001	103695000	2000	1	6.89E-06	-0.7201378	12	0.6	RELN	Protease
DMR7:110708001	7	110708001	110712000	4000	1	4.02E-08	-0.7197439	41	1.025	IMMP2L	Protease
DMR7:110843001	7	110843001	110845000	2000	1	6.88E-07	-0.7812022	15	0.75	IMMP2L	Protease
DMR7:130079001	7	130079001	130080000	1000	1	3.97E-06	0.5701097	13	1.3	KLHDC10	
DMR7:131148001	7	131148001	131150000	2000	1	3.71E-07	-0.6301175	19	0.95	MKLN1	Signaling
DMR7:133296001	7	133296001	133297000	1000	1	4.30E-06	-0.815133	3	0.3	EXOC4;MIR6133	Transport
DMR7:142062001	7	142062001	142063000	1000	1	5.33E-07	-0.8628877	12	1.2	MGAM	Metabolism
DMR7:142930001	7	142930001	142931000	1000	1	2.48E-06	-0.547	15	1.5	TRPV5;LLCFC1	Transport
DMR7:146096001	7	146096001	146097000	1000	1	2.61E-06	0.8492238	39	3.9		
DMR7:157874001	7	157874001	157875000	1000	1	1.39E-06	-0.3499685	27	2.7	PTPRN2;AC011899.3	Signaling
DMR8:482001	8	482001	483000	1000	1	4.98E-07	-0.6810516	17	1.7	FBXO25;AC083964.2;TDRP	
DMR8:1242001	8	1242001	1244000	2000	1	1.88E-07	-0.4707801	44	2.2	DLGAP2;AC110288.1	Receptor
DMR8:1824001	8	1824001	1826000	2000	2	5.56E-10	1.0155084	96	4.8	AC019257.8;MIR596;ARHGEF10	Protease
DMR8:5650001	8	5650001	5654000	4000	1	1.64E-07	-0.5336835	46	1.15	AC084768.1	
DMR8:6559001	8	6559001	6560000	1000	1	1.10E-06	0.8944343	14	1.4	MCPH1;ANGPT2	DNA Repair;Signaling
DMR8:7690001	8	7690001	7691000	1000	1	2.61E-07	1.0332821	14	1.4	AC084121.3;AC084121.4;AC084121.2	
DMR8:10430001	8	10430001	10432000	2000	1	2.86E-06	-0.4179805	33	1.65	MSRA;AC104964.3	Metabolism
DMR8:12623001	8	12623001	12625000	2000	1	2.45E-07	-1.1402102	29	1.45	AC068587.4;RPS3AP35	
DMR8:18621001	8	18621001	18623000	2000	1	5.96E-06	0.6731904	20	1	PSD3	Signaling
DMR8:22162001	8	22162001	22164000	2000	1	1.60E-06	-0.5803564	39	1.95	LGI3;SFTPC;BMP1;AC105206.1	Receptor;Unknown;Protease
DMR8:25681001	8	25681001	25683000	2000	1	1.05E-06	-0.4786134	10	0.5	AC009623.1	
DMR8:26563001	8	26563001	26565000	2000	1	8.10E-07	-0.3632575	14	0.7	DPYSL2	Metabolism
DMR8:35097001	8	35097001	35098000	1000	1	7.68E-06	-0.9679379	6	0.6		
DMR8:38869001	8	38869001	38870000	1000	1	3.10E-06	1.0914025	9	0.9		
DMR8:48530001	8	48530001	48531000	1000	1	2.53E-06	-0.9712279	6	0.6		
DMR8:51871001	8	51871001	51872000	1000	1	8.41E-06	-0.5646483	6	0.6	PCMTD1	Metabolism
DMR8:53301001	8	53301001	53302000	1000	1	8.87E-06	-0.8694708	5	0.5		
DMR8:59382001	8	59382001	59383000	1000	1	9.68E-08	-0.6404769	11	1.1		
DMR8:67401001	8	67401001	67402000	1000	1	2.11E-06	0.7281808	7	0.7	AC011037.1	
DMR8:71115001	8	71115001	71116000	1000	1	9.76E-06	0.8768569	13	1.3	AC015687.1	
DMR8:76198001	8	76198001	76199000	1000	1	3.52E-08	-0.6295675	5	0.5		
DMR8:81328001	8	81328001	81329000	1000	1	6.17E-07	-0.4740234	6	0.6		
DMR8:84728001	8	84728001	84730000	2000	1	1.43E-06	-0.5480921	10	0.5	RALYL	Transcription
DMR8:85216001	8	85216001	85217000	1000	1	1.62E-06	-0.5208883	7	0.7	E2F5;C8orf59;CA13;AC011773.3	Transcription;Metabolism
DMR8:86444001	8	86444001	86446000	2000	1	4.72E-07	-0.8015767	9	0.45	WWP1	Proteolysis
DMR8:88448001	8	88448001	88449000	1000	1	1.24E-06	0.8389684	10	1	AC090578.1	
DMR8:88646001	8	88646001	88647000	1000	1	9.72E-08	-0.8520037	3	0.3	AC090578.1	
DMR8:91548001	8	91548001	91550000	2000	1	1.28E-07	-1.1503793	21	1.05	AC103409.1	
DMR8:95873001	8	95873001	95875000	2000	1	8.32E-06	-0.6286364	8	0.4		
DMR8:96325001	8	96325001	96326000	1000	1	2.37E-09	-0.6699008	6	0.6	PTDSS1	Metabolism
DMR8:97331001	8	97331001	97332000	1000	1	9.10E-06	-0.5777146	10	1		
DMR8:114583001	8	114583001	114585000	2000	1	3.44E-07	-0.5416655	17	0.85		
DMR8:118551001	8	118551001	118553000	2000	1	6.80E-07	-0.5612178	17	0.85	SAMD12	Unknown
DMR8:118685001	8	118685001	118687000	2000	1	3.20E-07	-0.6557276	15	0.75	SAMD12-AS1	
DMR8:120190001	8	120190001	120192000	2000	1	8.49E-06	-0.5971808	10	0.5	COL14A1	Cytoskeleton
DMR8:122964001	8	122964001	122966000	2000	1	1.56E-06	-0.7544156	9	0.45	ZHX2	Transcription
DMR8:124750001	8	124750001	124751000	1000	1	5.93E-06	0.7510927	14	1.4		
DMR8:125639001	8	125639001	125641000	2000	1	7.56E-07	-0.5941291	27	1.35	AC016074.2	
DMR8:127990001	8	127990001	127991000	1000	1	3.65E-06	-0.5753967	7	0.7	PVT1;RNU1-106P	
DMR8:133091001	8	133091001	133093000	2000	1	1.34E-07	-0.4136718	30	1.5	TG;SLA	Signaling
DMR8:133250001	8	133250001	133251000	1000	1	6.01E-06	-0.6032632	18	1.8	NDRG1	Transcription
DMR8:136037001	8	136037001	136039000	2000	1	2.28E-07	1.0371973	23	1.15	LINC02055	
DMR8:142072001	8	142072001	142073000	1000	1	6.37E-07	0.8235722	13	1.3		
DMR9:24711001	9	24711001	24712000	1000	1	1.13E-06	-1.0081227	3	0.3		
DMR9:25308001	9	25308001	25310000	2000	1	5.33E-07	0.8622406	19	0.95		
DMR9:27371001	9	27371001	27372000	1000	1	6.39E-06	-0.4952696	11	1.1	MOB3B	Signaling
DMR9:31375001	9	31375001	31376000	1000	1	3.62E-08	-0.4665531	10	1	LINC01243	
DMR9:62708001	9	62708001	62710000	2000	1	4.99E-06	0.7495045	39	1.95	FKBP4P7	
DMR9:65560001	9	65560001	65561000	1000	1	7.15E-06	-0.6755017	7	0.7		
DMR9:72375001	9	72375001	72377000	2000	1	5.56E-07	0.6838577	33	1.65	ZFAND5	Transcription
DMR9:82141001	9	82141001	82142000	1000	1	3.98E-06	0.8524656	32	3.2	AL162726.3;AL158047.1	
DMR9:87814001	9	87814001	87816000	2000	1	1.99E-07	-0.9849743	9	0.45	AL772337.4;FBP2P1;ELF2P3;NPAP1P7	
DMR9:88286001	9	88286001	88287000	1000	1	3.02E-08	0.6938888	18	1.8		
DMR9:90190001	9	90190001	90192000	2000	1	9.63E-06	-1.0380804	7	0.35		

DMR9:90411001	9	90411001	90413000	2000	1	6.89E-06	-0.5865592	13	0.65	LINC01508	
DMR9:94745001	9	94745001	94747000	2000	1	3.85E-06	-0.5994547	27	1.35	C9orf3	Proteolysis
DMR9:98603001	9	98603001	98606000	3000	1	6.91E-06	-0.7573327	44	1.467	GABBR2;SEPT7P7	Receptor
DMR9:100217001	9	100217001	100218000	1000	1	9.55E-06	0.8744344	11	1.1	INVS	
DMR9:102024001	9	102024001	102027000	3000	1	6.18E-06	-0.8691895	11	0.367		
DMR9:116117001	9	116117001	116118000	1000	1	8.04E-07	-0.6406795	7	0.7		
DMR9:121919001	9	121919001	121920000	1000	1	9.38E-06	-0.6209179	4	0.4	TTLL11;TTLL11-IT1	Cytoskeleton
DMR9:124096001	9	124096001	124097000	1000	1	2.94E-06	0.5522826	18	1.8		
DMR9:125187001	9	125187001	125189000	2000	1	1.44E-06	0.8385809	58	2.9	PPP6C;RPSAP76	Signaling
DMR9:128010001	9	128010001	128012000	2000	1	9.26E-06	0.532175	31	1.55		
DMR9:128486001	9	128486001	128487000	1000	1	2.59E-06	-1.0404849	3	0.3	ODF2	Cytoskeleton
DMR9:129113001	9	129113001	129114000	1000	1	3.34E-07	0.587374	23	2.3	CRAT;PTPA	Metabolism
DMR9:134443001	9	134443001	134446000	3000	1	9.49E-07	0.9415373	53	1.767	RXRA	Receptor
DMR9:137140001	9	137140001	137142000	2000	1	1.23E-06	-0.7865738	51	2.55	GRIN1	Receptor
DMR10:5881001	10	5881001	5883000	2000	1	1.72E-06	0.645441	33	1.65	ANKRD16;FBH1	
DMR10:6852001	10	6852001	6853000	1000	1	7.94E-06	1.0386449	9	0.9	LINC00707;AL392086.2	
DMR10:6930001	10	6930001	6931000	1000	1	6.57E-06	-0.8284854	4	0.4	AL392086.1	
DMR10:7527001	10	7527001	7528000	1000	1	3.74E-06	-0.4612989	7	0.7	AL445070.1	
DMR10:19091001	10	19091001	19092000	1000	1	1.35E-07	-0.5344647	8	0.8	MALRD1	
DMR10:20290001	10	20290001	20292000	2000	1	2.52E-07	0.7460668	15	0.75	PLXDC2	Binding Protein
DMR10:20821001	10	20821001	20822000	1000	1	1.28E-09	-0.7634124	6	0.6	NEBL	Cytoskeleton
DMR10:26085001	10	26085001	26087000	2000	1	7.85E-06	-0.7895772	9	0.45	MYO3A	Cytoskeleton
DMR10:27491001	10	27491001	27492000	1000	1	1.51E-08	-0.6822888	7	0.7		
DMR10:27628001	10	27628001	27629000	1000	1	3.66E-06	-0.6216715	13	1.3		
DMR10:34836001	10	34836001	34837000	1000	1	7.09E-06	-0.3568398	15	1.5		
DMR10:37507001	10	37507001	37508000	1000	1	3.45E-06	1.0486368	16	1.6		
DMR10:43225001	10	43225001	43226000	1000	1	1.77E-06	-0.3470552	12	1.2	RASGEF1A	Signaling
DMR10:49630001	10	49630001	49633000	3000	1	2.75E-06	-0.3770004	37	1.233	CHAT	Metabolism
DMR10:49806001	10	49806001	49807000	1000	1	1.26E-06	0.7055622	18	1.8	RPL21P89	
DMR10:54518001	10	54518001	54519000	1000	1	9.94E-06	-0.4557692	9	0.9	PCDH15;AL353784.1	Extracellular Matrix
DMR10:57997001	10	57997001	57998000	1000	1	2.56E-06	-0.5250618	6	0.6		
DMR10:64949001	10	64949001	64951000	2000	1	9.17E-07	-0.5648882	6	0.3		
DMR10:69258001	10	69258001	69260000	2000	1	7.13E-07	-0.5566237	14	0.7	HKDC1;AL596223.2;HK1	Signaling
DMR10:70813001	10	70813001	70814000	1000	1	6.98E-06	-0.4802403	11	1.1	SGPL1	Metabolism
DMR10:72197001	10	72197001	72199000	2000	1	2.65E-06	0.5785468	22	1.1	ASCC1;RPL15P14	Binding Protein
DMR10:73625001	10	73625001	73626000	1000	1	1.86E-06	0.7193911	55	5.5	USP54;AC073389.1;AC073389.3;MYOZ1	
DMR10:76218001	10	76218001	76222000	4000	1	5.21E-06	-0.653641	30	0.75	LRMDA	
DMR10:77091001	10	77091001	77092000	1000	1	9.93E-07	-0.6685994	14	1.4	KCNMA1	Metabolism
DMR10:79033001	10	79033001	79035000	2000	1	1.90E-06	-0.2902581	32	1.6	ZMIZ1-AS1	
DMR10:79325001	10	79325001	79328000	3000	1	5.79E-06	-0.3867976	21	0.7	ZMIZ1	Metabolism
DMR10:86365001	10	86365001	86366000	1000	1	4.36E-07	0.767855	62	6.2	GRID1	Receptor
DMR10:91469001	10	91469001	91472000	3000	1	7.56E-06	-0.8092263	13	0.433	HECTD2;AL161798.1	EST
DMR10:100809001	10	100809001	100810000	1000	1	2.62E-06	0.6844549	15	1.5	PAX2	Transcription
DMR10:101619001	10	101619001	101622000	3000	1	1.67E-06	1.2099467	40	1.333	DPCD;FBXW4;RNU6-1165P	Unknown
DMR10:101661001	10	101661001	101663000	2000	1	4.42E-08	-0.5988523	19	0.95	FBXW4	Unknown
DMR10:103389001	10	103389001	103392000	3000	1	8.61E-06	0.6316328	44	1.467	TAF5;ATP5MD;MIR1307;PDCD11	Transcription;Apoptosis
DMR10:106795001	10	106795001	106796000	1000	1	4.54E-06	-0.6829713	7	0.7	SORCS1	Receptor
DMR10:113356001	10	113356001	113357000	1000	1	6.23E-06	-0.9072859	7	0.7	RNU7-165P	
DMR10:117499001	10	117499001	117501000	2000	1	8.78E-06	-0.6012907	30	1.5	AC005871.2;EMX2OS	
DMR10:118021001	10	118021001	118023000	2000	1	5.68E-08	-0.4882908	13	0.65	RAB11FIP2;AC022395.1	
DMR10:125943001	10	125943001	125944000	1000	1	4.47E-06	1.042737	12	1.2	FANK1	
DMR10:133012001	10	133012001	133013000	1000	1	7.74E-06	-0.4565435	49	4.9		
DMR11:3911001	11	3911001	3913000	2000	1	3.62E-06	0.633939	9	0.45	STIM1;RF00409	Signaling
DMR11:6281001	11	6281001	6282000	1000	1	9.32E-06	0.5672051	16	1.6	CCKBR	Receptor
DMR11:14276001	11	14276001	14278000	2000	1	5.87E-08	0.5808539	17	0.85	SPON1;SPON1-AS1;RRAS2	Growth Factors & Cytokines;Signaling
DMR11:19359001	11	19359001	19360000	1000	1	2.69E-08	-0.6964187	8	0.8	NAV2	Development
DMR11:22614001	11	22614001	22615000	1000	1	5.04E-07	-0.72362	5	0.5	FANCF	
DMR11:24281001	11	24281001	24283000	2000	1	4.83E-06	-0.5355246	17	0.85		
DMR11:24960001	11	24960001	24962000	2000	1	9.40E-07	-0.490419	11	0.55	LUZP2	
DMR11:31576001	11	31576001	31578000	2000	1	8.37E-06	-0.4835536	17	0.85	ELP4	Transcription
DMR11:33571001	11	33571001	33573000	2000	1	4.44E-06	0.7680501	21	1.05	KIAA1549L	
DMR11:35771001	11	35771001	35772000	1000	1	3.97E-07	0.5739109	14	1.4	TRIM44	
DMR11:40588001	11	40588001	40589000	1000	1	8.95E-06	-0.3503883	9	0.9	LRRC4C	Extracellular Matrix
DMR11:46022001	11	46022001	46025000	3000	1	6.14E-08	0.7306804	24	0.8	PHF21A	Metabolism
DMR11:49397001	11	49397001	49398000	1000	1	5.12E-06	-0.6419734	4	0.4	TYRL;CBX3P8	
DMR11:56338001	11	56338001	56340000	2000	1	7.61E-06	-0.816767	16	0.8	FAM8A2P;OR8K2P;OR8K1	Receptor
DMR11:61100001	11	61100001	61101000	1000	1	3.13E-06	0.6428472	2	0.2	CD5	Receptor
DMR11:62013001	11	62013001	62014000	1000	1	6.72E-06	-0.8931881	12	1.2	AP003733.1	

DMR11:63013001	11	63013001	63014000	1000	1	2.77E-06	-0.6064386	10	1	SLC22A8	Transport
DMR11:71288001	11	71288001	71289000	1000	1	5.82E-06	-0.6601629	7	0.7		
DMR11:72648001	11	72648001	72650000	2000	1	3.42E-07	-0.4059606	18	0.9	PDE2A;AP003065.1	Signaling
DMR11:76945001	11	76945001	76946000	1000	1	1.93E-06	-0.7038751	7	0.7	ACER3;AP002498.1	Metabolism
DMR11:78428001	11	78428001	78431000	3000	1	1.93E-06	-0.6269496	17	0.567	GAB2;AP003086.2;AP003086.1;NARS2	Receptor;Transcription
DMR11:79350001	11	79350001	79352000	2000	1	9.99E-07	-0.3244548	21	1.05	TENM4	Signaling
DMR11:79876001	11	79876001	79877000	1000	1	3.53E-07	1.447963	6	0.6		
DMR11:93175001	11	93175001	93176000	1000	1	9.74E-06	-0.42818	12	1.2	SLC36A4;AP003072.5	Transport
DMR11:94281001	11	94281001	94282000	1000	1	1.64E-07	-0.8646871	9	0.9	AP002784.1	
DMR11:95592001	11	95592001	95593000	1000	1	4.67E-07	-0.730889	7	0.7	AP000820.2	
DMR11:97733001	11	97733001	97737000	4000	1	3.85E-06	-0.6907272	10	0.25		
DMR11:106275001	11	106275001	106276000	1000	1	3.84E-06	-0.7619225	6	0.6		
DMR11:109564001	11	109564001	109565000	1000	1	6.72E-07	-0.6557736	2	0.2	AP003049.2	
DMR11:111872001	11	111872001	111873000	1000	1	4.27E-06	-0.6308238	8	0.8	ALG9;AP001781.2;GNG5P3;FDXACB1;C11orf1	Metabolism
DMR11:112401001	11	112401001	112403000	2000	1	2.73E-06	-0.4219308	18	0.9	AP003063.1	
DMR11:120177001	11	120177001	120179000	2000	1	3.39E-07	1.0832786	18	0.9	TRIM29;AP000679.1	Metabolism
DMR11:121501001	11	121501001	121502000	1000	1	4.35E-06	-0.7893128	6	0.6	SORL1	Receptor
DMR11:128972001	11	128972001	128973000	1000	1	1.59E-06	-0.5045185	10	1	ARHGAP32	Signaling
DMR12:4746001	12	4746001	4748000	2000	1	3.52E-07	0.697345	14	0.7	AC005833.1;GALNT8	Metabolism
DMR12:5142001	12	5142001	5143000	1000	1	3.22E-06	-0.4060174	17	1.7		
DMR12:7100001	12	7100001	7105000	5000	1	7.21E-06	-0.4409029	58	1.16	C1R;C1RL;C1RL-AS1	Immune;Protease
DMR12:9415001	12	9415001	9416000	1000	1	8.47E-06	0.4767515	21	2.1	AC141557.1;AC141557.2;DDX12P	
DMR12:9579001	12	9579001	9580000	1000	1	6.05E-06	-1.3024967	8	0.8	AC092821.3;AC092821.1	
DMR12:10651001	12	10651001	10653000	2000	1	1.93E-08	-0.7747836	15	0.75	STYK1	Receptor
DMR12:13564001	12	13564001	13565000	1000	1	5.08E-06	-0.9862011	35	3.5	GRIN2B	Receptor
DMR12:19669001	12	19669001	19670000	1000	1	2.29E-07	-0.8077415	3	0.3	AEBP2	Transcription
DMR12:20061001	12	20061001	20062000	1000	1	5.69E-06	-0.5184235	8	0.8	LINC02398	
DMR12:27229001	12	27229001	27230000	1000	1	9.20E-06	-0.4982085	3	0.3		
DMR12:32916001	12	32916001	32917000	1000	1	3.67E-06	-0.5129993	6	0.6		
DMR12:43584001	12	43584001	43585000	1000	1	1.56E-06	-0.5114725	10	1		
DMR12:47009001	12	47009001	47010000	1000	1	9.47E-06	-0.5548508	9	0.9		
DMR12:49758001	12	49758001	49759000	1000	1	1.67E-06	-0.6080902	10	1	TMBIM6	Apoptosis
DMR12:53498001	12	53498001	53500000	2000	1	1.79E-06	0.8645961	61	3.05	MAP3K12;AC023509.3;TARBP2;ATF7;NPFF	Signaling;Transcription
DMR12:55811001	12	55811001	55812000	1000	1	6.24E-06	-0.5246924	9	0.9	SARNP;AC023055.1;ORMDL2;DNAJC14	Transcription;Translation;Protein Binding
DMR12:62445001	12	62445001	62446000	1000	1	2.93E-07	-0.7140059	4	0.4		
DMR12:68739001	12	68739001	68740000	1000	1	6.63E-06	0.8115642	20	2	NUP107;SLC35E3	Transport
DMR12:71103001	12	71103001	71104000	1000	1	7.41E-06	1.1122277	6	0.6	AC025575.1;AC025575.2	
DMR12:75480001	12	75480001	75482000	2000	1	2.99E-06	-0.5648618	21	1.05	GLIPR1;AC121761.1;KRR1	Transcription
DMR12:81780001	12	81780001	81781000	1000	1	3.89E-06	-1.1812637	4	0.4		
DMR12:86917001	12	86917001	86919000	2000	1	5.12E-06	-0.6624892	13	0.65		
DMR12:89311001	12	89311001	89314000	3000	1	5.48E-06	0.6391671	34	1.133	LINC02458;MRPS6P4	
DMR12:99154001	12	99154001	99155000	1000	1	4.73E-06	-0.4861876	17	1.7	ANKS1B	Receptor
DMR12:101460001	12	101460001	101461000	1000	1	6.30E-06	0.5985655	16	1.6	RNU5E-5P	
DMR12:102110001	12	102110001	102112000	2000	1	8.81E-06	-0.9481755	13	0.65	NUP37;PARPBP	Metabolism
DMR12:102176001	12	102176001	102177000	1000	1	4.75E-06	0.7412656	9	0.9	PARPBP	
DMR12:102498001	12	102498001	102499000	1000	1	4.99E-06	-0.4957777	2	0.2		
DMR12:105630001	12	105630001	105632000	2000	1	2.23E-06	-0.4978292	15	0.75		
DMR12:106215001	12	106215001	106216000	1000	1	2.79E-06	0.4783249	19	1.9		
DMR12:109267001	12	109267001	109269000	2000	1	4.92E-06	-0.3766094	33	1.65	ACACB;FOXN4	Metabolism;Transcription
DMR12:110325001	12	110325001	110327000	2000	1	1.30E-07	-0.9564508	18	0.9	ATP2A2	Metabolism
DMR12:110336001	12	110336001	110337000	1000	1	5.90E-06	-0.7809265	7	0.7	ATP2A2	Metabolism
DMR12:110441001	12	110441001	110445000	4000	1	4.96E-07	0.7889969	75	1.875	AC144548.1;ARPC3;GPN3	Cytoskeleton;Transcription
DMR12:112178001	12	112178001	112179000	1000	1	3.32E-06	0.868337	21	2.1	HECTD4	
DMR12:114323001	12	114323001	114324000	1000	1	4.39E-06	-0.6522382	11	1.1		
DMR12:114875001	12	114875001	114879000	4000	1	3.57E-06	0.5413825	50	1.25		
DMR12:119809001	12	119809001	119810000	1000	1	3.51E-06	-0.5548171	11	1.1	CIT	Signaling
DMR12:121514001	12	121514001	121516000	2000	1	3.39E-07	0.8815815	34	1.7	KDM2B	
DMR12:123452001	12	123452001	123453000	1000	1	1.90E-06	-0.4962994	9	0.9	SNRNP35	Translation
DMR12:124005001	12	124005001	124006000	1000	1	8.23E-06	0.9747191	10	1	ZNF664;RFLNA;AC068790.8;AC068790.1	Transcription
DMR12:125102001	12	125102001	125104000	2000	1	1.21E-06	0.725551	42	2.1	AACS	Metabolism
DMR12:125211001	12	125211001	125212000	1000	1	4.67E-06	-0.6246062	9	0.9	TMEM132B	Unknown
DMR12:128045001	12	128045001	128046000	1000	1	5.88E-06	0.5920729	15	1.5		
DMR12:128609001	12	128609001	128610000	1000	1	5.10E-06	0.7145798	6	0.6	TMEM132C	Unknown
DMR12:128663001	12	128663001	128666000	3000	1	1.78E-06	-0.5331325	66	2.2	TMEM132C	Unknown
DMR12:128711001	12	128711001	128714000	3000	1	7.13E-06	0.7826136	51	1.7	TMEM132C	Unknown
DMR12:131432001	12	131432001	131436000	4000	1	8.59E-06	1.0151738	177	4.425	AC073578.4	
DMR12:132316001	12	132316001	132317000	1000	1	4.95E-06	-0.4315296	31	3.1	GALNT9	Metabolism

DMR12:132731001	12	132731001	132734000	3000	1	1.20E-06	0.6993745	151	5.033	PGAM5;RNA5SP379;ANKLE2	
DMR13:19292001	13	19292001	19293000	1000	1	2.74E-06	-0.6175243	12	1.2	ANKRD26P3	
DMR13:21960001	13	21960001	21962000	2000	1	9.31E-06	-0.6041032	13	0.65		
DMR13:26666001	13	26666001	26667000	1000	1	3.98E-06	0.5971087	16	1.6	WASF3	Cytoskeleton
DMR13:26944001	13	26944001	26945000	1000	1	3.80E-06	-0.5491039	5	0.5		
DMR13:28996001	13	28996001	28999000	3000	1	3.18E-07	-0.5977335	29	0.967	MTUS2	Cytoskeleton
DMR13:36497001	13	36497001	36498000	1000	1	3.79E-06	1.036701	5	0.5	HIST1H2APS6	
DMR13:44088001	13	44088001	44089000	1000	1	2.34E-06	-0.6920414	8	0.8		
DMR13:45282001	13	45282001	45284000	2000	1	2.40E-06	0.7220002	18	0.9	GTF2F2	Transcription
DMR13:56816001	13	56816001	56818000	2000	1	1.38E-06	-0.6806105	7	0.35		
DMR13:59560001	13	59560001	59561000	1000	1	6.94E-06	-0.5272228	3	0.3		
DMR13:59957001	13	59957001	59958000	1000	1	7.18E-07	-0.6158558	4	0.4	DIAPH3	Cytoskeleton
DMR13:60867001	13	60867001	60869000	2000	1	1.10E-08	-0.7810444	3	0.15		
DMR13:65751001	13	65751001	65752000	1000	1	1.98E-06	-0.9786599	2	0.2		
DMR13:67510001	13	67510001	67511000	1000	1	9.08E-06	-0.6254222	9	0.9		
DMR13:69426001	13	69426001	69430000	4000	1	5.58E-06	0.5709394	20	0.5		
DMR13:73229001	13	73229001	73231000	2000	1	3.07E-08	0.9013651	18	0.9	RNY1P8	
DMR13:75109001	13	75109001	75112000	3000	1	7.76E-06	-0.4875661	21	0.7	RNU6-38P	
DMR13:79970001	13	79970001	79972000	2000	1	1.69E-06	-0.6902922	11	0.55	AL158064.1	
DMR13:95689001	13	95689001	95690000	1000	1	2.64E-06	-0.4309865	4	0.4	DNAJC3;MTND5P2;MTND6P18;MTCYBP3	Protein Binding
DMR13:98266001	13	98266001	98267000	1000	1	6.40E-06	0.6814442	15	1.5	FARP1	Signaling
DMR13:99311001	13	99311001	99316000	5000	1	5.06E-07	-0.4679861	64	1.28	UBAC2;GPR183	Signaling
DMR13:105201001	13	105201001	105202000	1000	1	4.30E-07	-1.2078866	4	0.4		
DMR13:107931001	13	107931001	107932000	1000	1	4.44E-07	0.7650271	5	0.5		
DMR13:113470001	13	113470001	113471000	1000	1	2.93E-06	-0.5195481	15	1.5	DCUN1D2;DCUN1D2-AS;RNU1-16P	Proteolysis
DMR14:24027001	14	24027001	24028000	1000	1	8.41E-06	-0.5667869	3	0.3	AL136419.1;DHRS4L1	Metabolism
DMR14:24415001	14	24415001	24416000	1000	1	1.05E-06	-0.4544707	28	2.8	NYNRIN	Transcription
DMR14:26918001	14	26918001	26919000	1000	1	8.53E-06	-1.0542583	9	0.9	AL110292.1;MIR4307HG;MIR4307	
DMR14:27063001	14	27063001	27064000	1000	1	9.70E-06	-0.675709	12	1.2	AL110292.1	
DMR14:30687001	14	30687001	30689000	2000	1	4.95E-07	-0.5831677	17	0.85	SCFD1;UBE2CP1	Receptor
DMR14:34790001	14	34790001	34792000	2000	1	6.58E-06	0.6923428	19	0.95	BAZ1A	Metabolism
DMR14:48570001	14	48570001	48572000	2000	1	4.86E-06	-0.7430836	17	0.85		
DMR14:52035001	14	52035001	52037000	2000	1	2.24E-06	-0.6898251	19	0.95	NID2	Extracellular Matrix
DMR14:63549001	14	63549001	63551000	2000	1	4.26E-06	0.4715623	40	2	PPP2R5E;AL136038.3	Signaling
DMR14:66714001	14	66714001	66716000	2000	1	2.42E-06	-0.4104103	12	0.6	GPHN	Receptor
DMR14:67093001	14	67093001	67094000	1000	1	1.31E-06	-0.9221725	3	0.3	GPHN	Receptor
DMR14:69838001	14	69838001	69840000	2000	1	2.99E-07	0.5105971	14	0.7		
DMR14:76610001	14	76610001	76611000	1000	1	2.87E-07	1.1097524	6	0.6	AC008050.1	
DMR14:79029001	14	79029001	79031000	2000	1	3.07E-07	-0.7369459	10	0.5	NRXN3	Receptor
DMR14:80091001	14	80091001	80092000	1000	1	4.87E-06	-0.4813456	7	0.7		
DMR14:86746001	14	86746001	86748000	2000	1	2.02E-06	-0.6001955	11	0.55		
DMR14:88290001	14	88290001	88292000	2000	1	2.92E-06	-0.6365923	21	1.05	KCNK10	Transport
DMR14:90106001	14	90106001	90110000	4000	1	7.56E-07	0.6835915	76	1.9	KCNK13;GLRXP2	Metabolism
DMR14:90379001	14	90379001	90380000	1000	1	5.95E-06	-0.4452028	8	0.8	AL512791.2	
DMR14:92547001	14	92547001	92548000	1000	1	6.70E-06	0.9411342	6	0.6	RIN3	Signaling
DMR14:95446001	14	95446001	95448000	2000	1	3.08E-07	-0.5229343	30	1.5	SYNE3	
DMR14:96645001	14	96645001	96648000	3000	1	7.46E-07	-0.6205166	10	0.333	RN7SKP108	
DMR14:99448001	14	99448001	99449000	1000	1	1.12E-06	-0.4549786	13	1.3	SETD3;RNU6-91P	
DMR14:101827001	14	101827001	101828000	1000	1	9.70E-06	0.7856008	12	1.2	PPP2R5C;AL137779.1	Signaling
DMR14:103701001	14	103701001	103702000	1000	1	1.39E-06	0.4950851	36	3.6	KLC1;AL049840.2;AL049840.3;AL049840.4;XRCC3	Cytoskeleton;Transcription
DMR14:106189001	14	106189001	106190000	1000	1	3.29E-06	0.7841305	11	1.1	SLC20A1P2;IGHV1-18;IGHV3-19	
DMR15:30831001	15	30831001	30833000	2000	1	3.79E-07	-0.4597954	32	1.6	HERC2P10	
DMR15:34840001	15	34840001	34843000	3000	1	8.34E-06	-0.630889	23	0.767	AC018868.2;AQR	Transcription
DMR15:36684001	15	36684001	36688000	4000	1	6.49E-06	-0.4764924	26	0.65	C15orf41	EST
DMR15:40174001	15	40174001	40175000	1000	1	3.70E-07	0.7124244	14	1.4	BUB1B	Signaling
DMR15:40422001	15	40422001	40423000	1000	1	1.30E-06	0.6713205	48	4.8	IVD	Metabolism
DMR15:42047001	15	42047001	42048000	1000	1	6.07E-08	-0.8507646	8	0.8	PLA2G4E	Metabolism
DMR15:42282001	15	42282001	42283000	1000	1	3.90E-06	1.0894247	10	1	TMEM87A;GANC	Unknown;Metabolism
DMR15:43596001	15	43596001	43599000	3000	1	1.34E-06	-0.5614992	29	0.967	PPIP5K1;CKMT1B;STRC;RNU6-554P	Signaling;Extracellular Matrix
DMR15:44253001	15	44253001	44256000	3000	1	8.70E-06	-0.4641334	29	0.967		
DMR15:49400001	15	49400001	49401000	1000	1	5.26E-06	-0.6751511	5	0.5	FAM227B	
DMR15:52365001	15	52365001	52366000	1000	1	1.70E-06	-0.6316008	7	0.7	MYO5A	Cytoskeleton
DMR15:53743001	15	53743001	53744000	1000	1	5.22E-06	0.9684616	20	2	WDR72	
DMR15:61121001	15	61121001	61124000	3000	1	3.76E-06	-1.1658575	35	1.167	RORA	Receptor
DMR15:62743001	15	62743001	62744000	1000	1	6.34E-06	-0.8823453	9	0.9	TLN2	Cytoskeleton
DMR15:62963001	15	62963001	62966000	3000	1	9.65E-08	-0.6067509	39	1.3	AC079328.1	
DMR15:64230001	15	64230001	64231000	1000	1	4.83E-06	0.4355004	21	2.1	CSNK1G1;AC087632.1	Signaling
DMR15:66241001	15	66241001	66242000	1000	1	1.99E-09	-0.6412479	13	1.3	MEGF11	Extracellular Matrix

DMR15:68771001	15	68771001	68772000	1000	1	2.89E-06	-0.4701824	5	0.5	ANP32A	Signaling
DMR15:70433001	15	70433001	70434000	1000	1	1.79E-07	-0.5189136	18	1.8		
DMR15:70529001	15	70529001	70532000	3000	1	6.97E-06	-0.3902115	25	0.833		
DMR15:71213001	15	71213001	71214000	1000	1	8.58E-07	-1.1917199	6	0.6	THSD4	Extracellular Matrix
DMR15:75185001	15	75185001	75186000	1000	1	7.13E-06	0.5836484	17	1.7	RPL36AP45;C15orf39	Growth Factors & Cytokines
DMR15:77892001	15	77892001	77893000	1000	1	4.85E-06	0.5930174	16	1.6	CSPG4P13	
DMR15:77963001	15	77963001	77964000	1000	1	3.35E-06	-0.6939627	8	0.8	AC104758.5	
DMR15:78209001	15	78209001	78211000	2000	1	6.59E-06	0.5237414	36	1.8	ACSBG1	Metabolism
DMR15:78624001	15	78624001	78626000	2000	1	7.74E-06	-0.360405	29	1.45	CHRNA3;CHRNB4;AC067863.1	Receptor
DMR15:78920001	15	78920001	78921000	1000	1	3.45E-06	0.984213	8	0.8	CTSH	Protease
DMR15:86743001	15	86743001	86744000	1000	1	7.44E-06	-0.6118818	10	1	AGBL1	Signaling
DMR15:87023001	15	87023001	87026000	3000	1	8.07E-09	-0.7509167	9	0.3	AGBL1	Signaling
DMR15:91262001	15	91262001	91264000	2000	1	2.19E-06	-0.3980937	28	1.4	SV2B	Binding Protein
DMR15:98978001	15	98978001	98980000	2000	1	1.95E-06	0.7588471	34	1.7	AC036108.1;PGPEP1L	Protease
DMR15:101548001	15	101548001	101550000	2000	1	2.53E-06	-0.4412748	38	1.9		
DMR16:3791001	16	3791001	3792000	1000	1	8.93E-07	-0.4352327	24	2.4	CREBBP	Transcription
DMR16:5472001	16	5472001	5475000	3000	1	2.42E-06	0.5231262	35	1.167	RBFOX1	Unknown
DMR16:6397001	16	6397001	6399000	2000	1	4.39E-06	-0.8114704	16	0.8	RBFOX1	Unknown
DMR16:6851001	16	6851001	6852000	1000	1	4.59E-06	-0.563282	11	1.1	RBFOX1	Unknown
DMR16:6971001	16	6971001	6972000	1000	1	2.10E-06	-0.3482249	14	1.4	RBFOX1	Unknown
DMR16:11992001	16	11992001	11995000	3000	1	1.99E-06	0.5223881	37	1.233	SNX29	Cytoskeleton
DMR16:12633001	16	12633001	12634000	1000	1	4.43E-06	0.6979455	18	1.8		
DMR16:19052001	16	19052001	19053000	1000	1	1.12E-06	0.6511852	15	1.5	TMC7;AC099518.4	Transport
DMR16:24437001	16	24437001	24440000	3000	1	5.38E-06	-0.7692339	19	0.633		
DMR16:27243001	16	27243001	27244000	1000	1	4.77E-06	-0.4181808	16	1.6	NSMCE1	
DMR16:28616001	16	28616001	28617000	1000	1	1.97E-06	-0.4645907	22	2.2	SULT1A1;AC020765.5	Metabolism
DMR16:31116001	16	31116001	31117000	1000	1	1.98E-06	0.6873786	60	6	BCKDK;KAT8;AC135050.5;AC135050.6	Signaling;Epigenetic
DMR16:46538001	16	46538001	46540000	2000	1	5.53E-06	-0.4745983	13	0.65	ANKRD26P1	
DMR16:55737001	16	55737001	55738000	1000	1	3.91E-06	-0.4457135	7	0.7	CES1P2	
DMR16:56968001	16	56968001	56969000	1000	1	3.35E-06	-0.4016815	14	1.4	CETP	Binding Protein
DMR16:66282001	16	66282001	66283000	1000	1	8.56E-07	-0.9281135	4	0.4		
DMR16:68303001	16	68303001	68305000	2000	1	1.43E-06	0.642697	29	1.45	SLC7A6;SLC7A6OS;PRMT7	Metabolism
DMR16:75102001	16	75102001	75103000	1000	1	8.16E-06	-0.3433842	21	2.1	ZNRF1;AC099508.1;LDHD	Transcription;Metabolism
DMR16:75181001	16	75181001	75182000	1000	1	4.54E-06	-0.7466281	6	0.6	ZFP1	
DMR16:76252001	16	76252001	76255000	3000	1	4.04E-06	-0.4643694	35	1.167	AC010528.1	
DMR16:77339001	16	77339001	77340000	1000	1	8.60E-07	-0.785832	8	0.8	ADAMTS18	Protease
DMR16:87824001	16	87824001	87825000	1000	1	3.42E-06	-0.5329661	20	2	SLC7A5;MIR6775	Metabolism
DMR16:87970001	16	87970001	87973000	3000	1	5.79E-06	-0.3894862	42	1.4	BANP	Transcription
DMR16:89402001	16	89402001	89403000	1000	1	5.94E-06	0.4520241	28	2.8	ANKRD11	EST
DMR16:89713001	16	89713001	89714000	1000	1	4.47E-06	0.5205058	32	3.2	VPS9D1;VPS9D1-AS1;ZNF276	Transcription
DMR17:963001	17	963001	964000	1000	1	8.05E-10	0.5073656	27	2.7	NXN;AC036164.1	Signaling
DMR17:6433001	17	6433001	6434000	1000	1	2.06E-06	-0.3925399	25	2.5	AIPL1	Protein Binding
DMR17:6984001	17	6984001	6985000	1000	1	1.41E-06	0.6759975	13	1.3	ALOX12-AS1;AC027763.2;AC040977.2;AC040977.1	
DMR17:7838001	17	7838001	7839000	1000	1	2.42E-07	1.1202783	12	1.2	DNAH2;KDM6B	Cytoskeleton;Transcription
DMR17:8909001	17	8909001	8911000	2000	1	1.70E-06	-0.5784843	34	1.7	PIK3R5	Signaling
DMR17:9707001	17	9707001	9708000	1000	1	9.47E-06	0.6569972	22	2.2	USP43	
DMR17:10595001	17	10595001	10597000	2000	1	5.43E-06	0.8541415	19	0.95	MYHAS;AC005323.1	
DMR17:11763001	17	11763001	11765000	2000	1	1.26E-08	-0.736115	21	1.05	DNAH9	Cytoskeleton
DMR17:11863001	17	11863001	11865000	2000	1	3.77E-07	-0.5703788	13	0.65	DNAH9;AC005209.1	Cytoskeleton
DMR17:16245001	17	16245001	16246000	1000	1	7.66E-06	0.4462797	23	2.3	PIGL	Metabolism
DMR17:18278001	17	18278001	18280000	2000	1	1.94E-06	0.5858592	38	1.9	AC127537.1;TOP3A	Transcription
DMR17:28031001	17	28031001	28033000	2000	1	4.47E-06	-0.6934768	18	0.9	AC090287.1;RF00156;NLK	Signaling
DMR17:30727001	17	30727001	30728000	1000	1	7.92E-07	-0.4150466	20	2	SUZ12P1;AC127024.7;AC127024.3;AC127024.2	
DMR17:33222001	17	33222001	33223000	1000	1	1.04E-06	-0.8983157	1	0.1	ASIC2	Transport
DMR17:34755001	17	34755001	34756000	1000	1	2.17E-06	-0.4877424	12	1.2		
DMR17:35682001	17	35682001	35684000	2000	1	6.58E-06	0.9483375	25	1.25	AP2B1	Transport
DMR17:36222001	17	36222001	36224000	2000	1	7.79E-09	0.9564993	26	1.3	CCL4L2;AC243829.5	
DMR17:36740001	17	36740001	36741000	1000	1	4.32E-07	-0.4195057	18	1.8		
DMR17:40506001	17	40506001	40508000	2000	1	8.91E-06	0.634816	18	0.9	TNS4;AC004585.1	Signaling
DMR17:40846001	17	40846001	40847000	1000	1	4.02E-06	-0.4223231	8	0.8	TMEM99;AC004231.3	
DMR17:42436001	17	42436001	42439000	3000	1	3.26E-06	0.5821806	46	1.533	RNU7-97P	
DMR17:43109001	17	43109001	43112000	3000	1	3.20E-06	0.5820137	54	1.8	BRCA1	Transcription
DMR17:44496001	17	44496001	44497000	1000	1	2.48E-06	0.8977599	12	1.2	GPATCH8;AC103703.1	
DMR17:50774001	17	50774001	50776000	2000	1	3.92E-07	0.6612705	43	2.15	ANKRD40CL;MIR8059;AC005921.4	
DMR17:55556001	17	55556001	55558000	2000	1	7.06E-07	-0.5026078	24	1.2	AC105021.1;GARSP1;RNU6-1249P	
DMR17:60736001	17	60736001	60738000	2000	1	2.22E-07	0.5793683	19	0.95	BCAS3;AC110602.1	Transcription
DMR17:61238001	17	61238001	61239000	1000	1	1.97E-06	0.8992406	19	1.9	BCAS3	Transcription

DMR17:63464001	17	63464001	63465000	1000	1	2.09E-06	-0.6950074	5	0.5	AC005828.5;PPIAP55	
DMR17:64998001	17	64998001	64999000	1000	1	2.28E-06	0.494202	18	1.8		
DMR17:67168001	17	67168001	67169000	1000	1	8.02E-07	0.7367953	11	1.1	HELZ	Transcription
DMR17:67631001	17	67631001	67634000	3000	1	7.11E-07	-0.5059349	61	2.033	PITPNC1;AC079331.1	
DMR17:68913001	17	68913001	68914000	1000	1	1.15E-08	-0.6930886	6	0.6	ABCA8	Receptor
DMR17:73378001	17	73378001	73379000	1000	1	1.80E-06	0.5528568	16	1.6	SDK2	Development
DMR17:73741001	17	73741001	73742000	1000	1	2.61E-06	-0.4850082	19	1.9	AC125421.1;LINC00469	
DMR17:75194001	17	75194001	75195000	1000	1	4.19E-06	0.5828536	45	4.5		
DMR17:75537001	17	75537001	75538000	1000	1	3.32E-07	0.621861	18	1.8	LLGL2	Development
DMR17:76900001	17	76900001	76902000	2000	1	4.88E-06	-0.3985727	36	1.8	MGAT5B	Metabolism
DMR17:77598001	17	77598001	77599000	1000	1	8.09E-06	0.7931789	16	1.6	AC021683.5	
DMR17:78336001	17	78336001	78337000	1000	1	4.25E-06	0.5847373	15	1.5	AC087645.2	
DMR18:3760001	18	3760001	3762000	2000	1	9.46E-06	-0.4058592	28	1.4	DLGAP1;AP002478.2	Signaling
DMR18:8749001	18	8749001	8750000	1000	1	3.17E-06	-0.5126872	11	1.1	MTCL1	
DMR18:9902001	18	9902001	9903000	1000	1	5.82E-06	0.4980064	23	2.3	AC006238.1	
DMR18:10568001	18	10568001	10569000	1000	1	5.04E-07	1.172583	10	1		
DMR18:21429001	18	21429001	21430000	1000	1	4.35E-06	0.6792457	5	0.5	GREB1L;AC015878.1	
DMR18:22336001	18	22336001	22337000	1000	1	1.66E-08	-0.5445338	17	1.7		
DMR18:26821001	18	26821001	26822000	1000	1	1.04E-06	1.0010425	2	0.2	AQP4-AS1;AC018371.1;AC018371.2	
DMR18:31938001	18	31938001	31939000	1000	1	1.18E-06	1.0044881	5	0.5	TRAPPC8;RNU6-1050P;AC009831.1	
DMR18:35725001	18	35725001	35727000	2000	1	1.72E-06	-0.5902583	8	0.4	AC090229.1	
DMR18:55250001	18	55250001	55252000	2000	1	7.32E-06	-0.6984778	24	1.2	TCF4	Transcription
DMR18:56496001	18	56496001	56498000	2000	1	3.03E-06	-0.5625773	14	0.7		
DMR18:56968001	18	56968001	56969000	1000	1	5.66E-06	-0.4509659	5	0.5	WDR7	Unknown
DMR18:58343001	18	58343001	58344000	1000	1	4.45E-06	0.6257183	15	1.5	NEDD4L	Protease
DMR18:62436001	18	62436001	62438000	2000	1	6.86E-06	-0.7114954	34	1.7	ACTBP9	
DMR18:63599001	18	63599001	63601000	2000	1	9.61E-06	-0.8856543	15	0.75	SERPINB13	Protease
DMR18:67261001	18	67261001	67263000	2000	1	7.94E-07	-0.7200381	13	0.65		
DMR18:67302001	18	67302001	67303000	1000	1	6.31E-07	-1.1893104	6	0.6		
DMR18:73132001	18	73132001	73133000	1000	1	1.40E-06	-0.5854772	3	0.3		
DMR18:74204001	18	74204001	74205000	1000	1	4.98E-06	0.8467968	10	1	AC090398.1	
DMR18:78053001	18	78053001	78054000	1000	1	5.01E-06	-0.4758	17	1.7		
DMR19:603001	19	603001	605000	2000	1	6.20E-06	0.7706511	75	3.75	HCN2	Metabolism
DMR19:862001	19	862001	864000	2000	1	7.70E-06	0.8872191	103	5.15	ELANE;CFD;MED16	Protease;Immune;Transcriptio n
DMR19:3230001	19	3230001	3231000	1000	1	6.08E-08	-0.5573396	10	1	CELF5	Translation
DMR19:7500001	19	7500001	7504000	4000	1	7.11E-06	0.6057991	151	3.775	PEX11G;TEX45;AC008878.1	
DMR19:7599001	19	7599001	7601000	2000	1	4.15E-06	0.6055752	22	1.1	CAMSAP3	
DMR19:7830001	19	7830001	7831000	1000	1	9.26E-06	0.6855874	85	8.5	EVI5L	
DMR19:10197001	19	10197001	10199000	2000	1	3.21E-06	0.7194797	31	1.55	DNMT1	Epigenetic
DMR19:13626001	19	13626001	13627000	1000	1	6.20E-08	0.5860472	15	1.5	CACNA1A	Transport
DMR19:13800001	19	13800001	13801000	1000	1	7.08E-06	0.4843254	41	4.1	ZSWIM4;AC020916.2	Transcription
DMR19:16948001	19	16948001	16950000	2000	1	2.06E-07	0.7652186	21	1.05	CPAMD8	
DMR19:18085001	19	18085001	18087000	2000	1	1.27E-06	-0.5950983	29	1.45	IL12RB1	
DMR19:21043001	19	21043001	21044000	1000	1	4.89E-06	-0.427829	7	0.7	ZNF430	Transcription
DMR19:27955001	19	27955001	27956000	1000	1	8.25E-06	-0.5328916	6	0.6	AC006504.7;AC005357.2	
DMR19:29076001	19	29076001	29078000	2000	1	5.28E-06	-0.2891072	27	1.35		
DMR19:31144001	19	31144001	31145000	1000	1	1.69E-06	-0.4941799	8	0.8	AC020912.1;TSHZ3	Transcription
DMR19:33107001	19	33107001	33110000	3000	1	7.18E-06	-0.4886915	60	2	GPATCH1	
DMR19:36978001	19	36978001	36980000	2000	1	8.79E-06	-0.5872276	23	1.15	ZNF568	Transcription
DMR19:39861001	19	39861001	39862000	1000	1	1.19E-07	0.7759882	8	0.8	FCGBP	Extracellular Matrix
DMR19:42889001	19	42889001	42891000	2000	1	1.02E-06	-0.5300016	34	1.7	PSG1	Extracellular Matrix
DMR19:44922001	19	44922001	44924000	2000	1	9.88E-06	0.4064002	42	2.1	AC011481.4;APOC1;APOC1P1	Transport
DMR19:46318001	19	46318001	46319000	1000	1	1.24E-07	0.8315254	15	1.5	HIF3A;AC007193.2	Transcription
DMR19:50906001	19	50906001	50907000	1000	1	6.16E-06	0.6470385	8	0.8	KLKP1;KLK4	Protease
DMR20:9582001	20	9582001	9583000	1000	1	6.46E-06	-0.7489359	6	0.6	PAK5;AL353612.1	
DMR20:11768001	20	11768001	11770000	2000	1	1.93E-06	-0.6202546	14	0.7		
DMR20:13703001	20	13703001	13704000	1000	1	4.88E-08	0.8215851	13	1.3		
DMR20:15075001	20	15075001	15076000	1000	1	4.59E-06	-0.6158566	7	0.7	MACROD2	
DMR20:18989001	20	18989001	18990000	1000	1	5.57E-06	-0.8119199	7	0.7		
DMR20:22597001	20	22597001	22600000	3000	1	1.82E-06	-0.5801319	29	0.967	LNCNEF	
DMR20:23455001	20	23455001	23456000	1000	1	3.68E-07	-0.587544	11	1.1	CST11;RF00019;AL109954.2	Signaling
DMR20:26117001	20	26117001	26118000	1000	1	7.19E-06	-0.4663103	12	1.2	NCOR1P1	
DMR20:34042001	20	34042001	34043000	1000	1	9.08E-06	0.4914578	16	1.6	RALY;MIR4755	Transcription
DMR20:36082001	20	36082001	36084000	2000	1	5.12E-06	0.8193762	22	1.1	AL035420.1;HMGB3P2;AL035420.2;EPB 41L1	
DMR20:39079001	20	39079001	39080000	1000	1	3.50E-06	0.9926674	17	1.7		
DMR20:47404001	20	47404001	47405000	1000	1	1.44E-06	0.5621608	14	1.4	LINC01754	
DMR20:47941001	20	47941001	47942000	1000	1	2.38E-07	-0.484401	21	2.1	AL357558.2	
DMR20:48875001	20	48875001	48877000	2000	1	3.41E-06	-0.6567095	32	1.6		

DMR20:52445001	20	52445001	52447000	2000	1	1.88E-06	-0.3016192	23	1.15	LINC01524;AL109610.1	
DMR20:53996001	20	53996001	53997000	1000	1	2.25E-07	-0.4277728	11	1.1	BCAS1	
DMR20:55763001	20	55763001	55764000	1000	1	5.23E-06	-0.5593399	7	0.7		
DMR20:55944001	20	55944001	55945000	1000	1	2.32E-06	1.1446512	8	0.8		
DMR20:61483001	20	61483001	61484000	1000	1	4.29E-08	-0.5607195	16	1.6	CDH4	Extracellular Matrix
DMR20:64093001	20	64093001	64095000	2000	1	4.08E-06	0.8474669	26	1.3	OPRL1;LKAAEAR1;MYT1	Receptor;Transcription
DMR21:6152001	21	6152001	6153000	1000	1	8.97E-06	-0.4006685	21	2.1		
DMR21:6342001	21	6342001	6344000	2000	1	9.96E-06	-0.5381794	17	0.85	CU633906.2	
DMR21:10741001	21	10741001	10743000	2000	1	6.11E-06	-0.5337132	16	0.8		
DMR21:17965001	21	17965001	17966000	1000	1	2.16E-06	0.6491482	4	0.4	CHODL	
DMR21:19680001	21	19680001	19681000	1000	1	5.08E-07	-0.7726042	5	0.5		
DMR21:23640001	21	23640001	23641000	1000	1	2.15E-06	-0.9618059	6	0.6		
DMR21:23835001	21	23835001	23836000	1000	1	3.71E-08	1.1225167	11	1.1		
DMR21:26824001	21	26824001	26825000	1000	1	2.31E-06	0.8413207	26	2.6		
DMR21:31508001	21	31508001	31509000	1000	1	8.63E-06	-0.4623201	19	1.9	TIAM1	Transcription
DMR21:33493001	21	33493001	33494000	1000	1	5.79E-06	0.5975765	29	2.9	AP000302.1;DNAJC28;GART	Transcription;Metabolism
DMR21:38356001	21	38356001	38357000	1000	1	9.45E-08	-0.4944057	22	2.2		
DMR21:43583001	21	43583001	43585000	2000	1	3.04E-07	-0.5202146	12	0.6	HSF2BP	
DMR21:46148001	21	46148001	46149000	1000	1	5.34E-07	-0.5375053	17	1.7	FTCD;FTCD-AS1	
DMR22:17210001	22	17210001	17212000	2000	1	8.66E-06	0.4740007	51	2.55	ADA2;FAM32BP	Transcription
DMR22:18386001	22	18386001	18388000	2000	1	5.01E-06	0.7548866	11	0.55	FAM230J	
DMR22:29607001	22	29607001	29609000	2000	1	9.53E-06	0.4201027	30	1.5	NF2;RPEP4	Cytoskeleton
DMR22:34327001	22	34327001	34328000	1000	1	1.63E-06	-1.0055776	4	0.4		
DMR22:39704001	22	39704001	39706000	2000	1	3.50E-07	0.5982911	49	2.45		
DMR22:46325001	22	46325001	46329000	4000	1	6.89E-06	0.428583	91	2.275	GTSE1;TRMU	
DMR22:49957001	22	49957001	49958000	1000	1	5.09E-07	-0.4575814	23	2.3	PIM3;MIR6821	Epigenetic
DMRX:1364001	X	1364001	1369000	5000	1	4.56E-06	0.9289401	494	9.88	IL3RA	Receptor
DMRX:13651001	X	13651001	13653000	2000	1	6.31E-06	1.0192184	30	1.5	TCEANC	Transcription
DMRX:16276001	X	16276001	16277000	1000	1	5.26E-06	-0.6609142	4	0.4		
DMRX:17491001	X	17491001	17492000	1000	1	3.02E-07	-0.6759206	10	1	NHS	
DMRX:22781001	X	22781001	22782000	1000	1	7.68E-06	-0.8046573	5	0.5	PTCHD1-AS	
DMRX:23695001	X	23695001	23697000	2000	1	2.79E-06	0.8706407	42	2.1	PRDX4;ACOT9	Electron Transport;Metabolism
DMRX:24165001	X	24165001	24166000	1000	1	2.98E-06	0.9300575	13	1.3	ZFX	Transcription
DMRX:31900001	X	31900001	31901000	1000	1	3.63E-06	-0.9599591	13	1.3	DMD	Development
DMRX:44792001	X	44792001	44793000	1000	1	5.94E-06	0.6076124	11	1.1		
DMRX:46370001	X	46370001	46372000	2000	1	3.53E-06	1.0238952	34	1.7		
DMRX:46581001	X	46581001	46582000	1000	1	2.05E-08	0.6105555	27	2.7	CHST7	Metabolism
DMRX:51310001	X	51310001	51312000	2000	1	9.77E-06	-0.9422977	18	0.9		
DMRX:52488001	X	52488001	52489000	1000	1	2.93E-06	-0.5199447	18	1.8	BX510359.8;BX510359.7;RBM22P6;XAGE1A	
DMRX:53469001	X	53469001	53470000	1000	1	8.43E-06	0.9332251	7	0.7	VTRNA3-1P	
DMRX:72115001	X	72115001	72116000	1000	1	1.31E-06	-0.8874394	13	1.3	NHSL2	
DMRX:73379001	X	73379001	73380000	1000	1	9.24E-06	-0.8777041	5	0.5		
DMRX:91549001	X	91549001	91550000	1000	1	3.45E-06	0.8262897	21	2.1		
DMRX:97607001	X	97607001	97609000	2000	1	2.72E-06	-0.7725409	24	1.2	DIAPH2;DIAPH2-AS1	Cytoskeleton
DMRX:118476001	X	118476001	118479000	3000	1	3.48E-07	0.7506308	56	1.867		
DMRX:123148001	X	123148001	123149000	1000	1	8.23E-06	0.9915717	5	0.5		
DMRX:123827001	X	123827001	123831000	4000	1	3.22E-06	0.5590727	108	2.7		
DMRX:125538001	X	125538001	125539000	1000	1	4.60E-07	-1.1559361	3	0.3		
DMRX:127483001	X	127483001	127484000	1000	1	1.80E-06	-1.0343179	7	0.7		
DMRX:130717001	X	130717001	130718000	1000	1	1.53E-06	-0.4949376	5	0.5	ENOX2	Transcription
DMRX:135215001	X	135215001	135216000	1000	1	9.76E-06	-0.939075	13	1.3	AC234771.2	
DMRX:141265001	X	141265001	141266000	1000	1	1.11E-08	0.9501535	14	1.4	RBMX2P2	
DMRX:151196001	X	151196001	151197000	1000	1	7.27E-06	-1.0056685	6	0.6		
DMRX:155134001	X	155134001	155135000	1000	1	1.52E-08	-1.1075893	6	0.6	BX293995.1;MTCP1	
DMRX:155830001	X	155830001	155832000	2000	1	2.05E-07	-0.5866864	13	0.65	AMD1P2	
DMRY:26328001	Y	26328001	26329000	1000	1	7.16E-06	0.7192894	36	3.6	PPP1R12BP1	
