# Supplementary material for: Sperm DNA methylation epimutation biomarker for paternal offspring autism susceptibility
Source: Clin Epigenetics. 2021 Jan 7;13:6. doi: 10.1186/s13148-020-00995-2 (PMC7789568; doi:10.1186/s13148-020-00995-2)
Supplement: Supplementary file 5 — Additional file 5: Table S2. Gene and protein regulators of autism. The DMR-associated autism-related genes with symbol, description, and relevant references PubMed ID (PMID) numbers. [file 13148_2020_995_MOESM5_ESM.pdf]

Supplemental Table S2

DMR Associated Gene and Protein Regulators of Autism

Gene Name	Gene Description	Literature References PMID
SLC25A12	solute carrier family 25 member 12	19913066; 16205742; 19360665; 19913066; 18180767
NRXN3	neurexin 3	23306218
SEMA3F	semaphorin 3F	30635860
RORA	RAR related orphan receptor A	27179922; 26625251; 1336
RBFOX1	RNA binding fox-1 homolog 1	18329129; 17503474
CD5	CD5 molecule	28979127
CCR5	C-C motif chemokine receptor 5 (gene/	28986277
GRIN1	glutamate ionotropic receptor NMDA ty	31299220
GPHN	gephyrin	25149987
SLC7A5	solute carrier family 7 member 5	27912058
SNTG2	syntrophin gamma 2	17292328; 17292328
RELN	reelin	15749247; 15560956; 19359144; 25450950; 28966264; 26285919; 28966264; 15820235; 12192627
ARHGAP32	Rho GTPase activating protein 32	30045817
OPRM1	opioid receptor mu 1	21525276
DLGAP2	DLG associated protein 2	28407363
DIAPH3	diaphanous related formin 3	20308993; 20308993
CACNA1A	calcium voltage-gated channel subunit	28799511; 26566276; 26566276; 25735478; 26566276
KCNMA1	potassium calcium-activated channel su	17236127
TSHZ3	teashirt zinc finger homeobox 3	27668656
